# Key bacterial taxa and metabolic pathways affecting gut short-chain fatty acid profiles in early life

**DOI:** 10.1038/s41396-021-00937-7

**Published:** 2021-03-15

**Authors:** Naoki Tsukuda, Kana Yahagi, Taeko Hara, Yohei Watanabe, Hoshitaka Matsumoto, Hiroshi Mori, Koichi Higashi, Hirokazu Tsuji, Satoshi Matsumoto, Ken Kurokawa, Takahiro Matsuki

**Affiliations:** 1 Yakult Central Institute, Kunitachi, Tokyo Japan; 2 National Institute of Genetics, Mishima, Shizuoka Japan

**Keywords:** Microbial ecology, Microbiome, Bacterial genetics

## Abstract

Infant gut microbiota development affects the host physiology throughout life, and short-chain fatty acids (SCFAs) are promising key metabolites mediating microbiota-host relationships. Here, we investigated dense longitudinally collected faecal samples from 12 subjects during the first 2 years (*n* = 1048) to identify early life gut SCFA patterns and their relationships with the microbiota. Our results revealed three distinct phases of progression in the SCFA profiles: early phase characterised by low acetate and high succinate, middle-phase characterised by high lactate and formate and late-phase characterised by high propionate and butyrate. Assessment of the SCFA–microbiota relationships revealed that faecal butyrate is associated with increased Clostridiales and breastfeeding cessation, and that diverse and personalised assemblage of Clostridiales species possessing the acetyl-CoA pathway play major roles in gut butyrate production. We also found an association between gut formate and some infant-type bifidobacterial species, and that human milk oligosaccharides (HMO)-derived fucose is the substrate for formate production during breastfeeding. We identified genes upregulated in fucose and fucosylated HMO utilisation in infant-type bifidobacteria. Notably, bifidobacteria showed interspecific and intraspecific variation in the gene repertoires, and cross-feeding of fucose contributed to gut formate production. This study provides an insight into early life SCFA–microbiota relationships, which is an important step for developing strategies for modulating lifelong health.

## Introduction

Microbial colonisation in the human gut begins shortly after birth. Studies have shown that initial gut microbiota have remarkably distinct profiles from those of adults, widely differ among individuals, and their diversity in composition and function increases over the first few years of life [1]. Previous studies have shown the development of the gut microbiome in early life to be influenced by environmental factors, including delivery mode, feeding and antibiotic exposure [2, 3]. Breastfeeding has also been associated with the development of *Bifidobacterium*-dominant microbiota, and recent studies have emphasised the importance of microbial genes for the utilisation of human milk oligosaccharides (HMO) [4, 5].

Development of the initial gut microbiota has immediate and prolonged consequences on host health. Accumulating evidence in mice has demonstrated early life microbiota to be associated with enteropathogen susceptibility [6], fat accumulation [7] and immune system development [8]. Emerging data on human microbiota have revealed that delayed or altered establishment of the infant gut microbiota is associated with subsequent adiposity [9], risk of asthma [10, 11], onset of type 1 diabetes [12–14] and malnutrition [15].

The mechanisms by which the gut bacterial community affects host physiology have received much attention, and accumulating data have demonstrated short-chain fatty acids (SCFAs) to be the key metabolites mediating the symbiotic relationship [16–19]. SCFAs are produced mainly through the fermentation of microbiota-accessible carbohydrates [20], and the major gut SCFAs have been recognised to be acetate, propionate and butyrate (typically corresponding to 90–95% of total SCFA in a 3:1:1 ratio) [16–18]. Previous studies have reported that these SCFAs exhibit differences in organ distribution and physiological effects [19].

Butyrate serves as the primary energy source for gut colonocytes and is locally consumed. Butyrate inhibits histone deacetylases and activates G protein-coupled receptors, which exert various physiological effects [21, 22]. Acetate is the most abundant SCFA not only in the gut lumen but also in peripheral circulation [23], and can mediate fat accumulation via the GPR43 signalling pathway [24] and/or affecting appetite via a central homoeostatic mechanism [25]. Propionate is transferred to the liver to be used as a substrate for gluconeogenesis and exert several physiological functions [19, 26]. On the other hand, the effects of other gut microbiota-derived metabolites (e.g., formate and succinate) and short-chain hydroxyl-fatty acid (i.e., lactate, denoted as one of the SCFAs in this study) are still largely unknown [16–19].

Consistent with the results obtained using animal models or in vitro cultured cells, recent human studies have highlighted the importance of these microbiota-derived SCFAs. Cohort studies in adults have shown that bacterial taxa having genes for butyrate production are less abundant in patients with colorectal cancer [16, 27] and type 2 diabetes [28, 29]. A more recent study in infants has reported that subjects who developed type 1 diabetes or islet autoimmunity later in life had fewer genes for carbohydrate fermentation and SCFA production [13]. However, most associations between gut SCFA and risk of disease are based on the functional potential of the gut microbiota, and only a few human cohort studies have devoted efforts to investigating the concentrations of the key metabolites mediating this symbiotic relationship [11]. At present, the initial gut SCFA pattern, dynamics and equilibria are still not sufficiently characterised, and the detailed relationship with the gut microbiota remains largely unexplored.

In this study, we investigated the gut microbiome and their metabolite profiles of 1048 samples from 12 healthy term infants during the first 2 years of life. We further evaluated the association among SCFA profiles, gut microbiota composition and life events and tried to identify key bacterial lineages, genetic factors (metabolic pathways) and environmental factors that affect gut SCFA profiles in early life.

## Results

### Gut microbiota development in early life

To investigate the dynamics of gut microbiota and their metabolites in early life, we collected 1048 faecal samples from 12 full-term infants during their first 2 years of life. Infant stool samples were collected every day during the 1st week, every other day until the 1st month, every week until 1 year of age and every other week thereafter (up to 92 stool samples per infant). All infants were Japanese, delivered vaginally and fed breast milk as the dominant nutrient (Table S1).

**Fig. 1:**
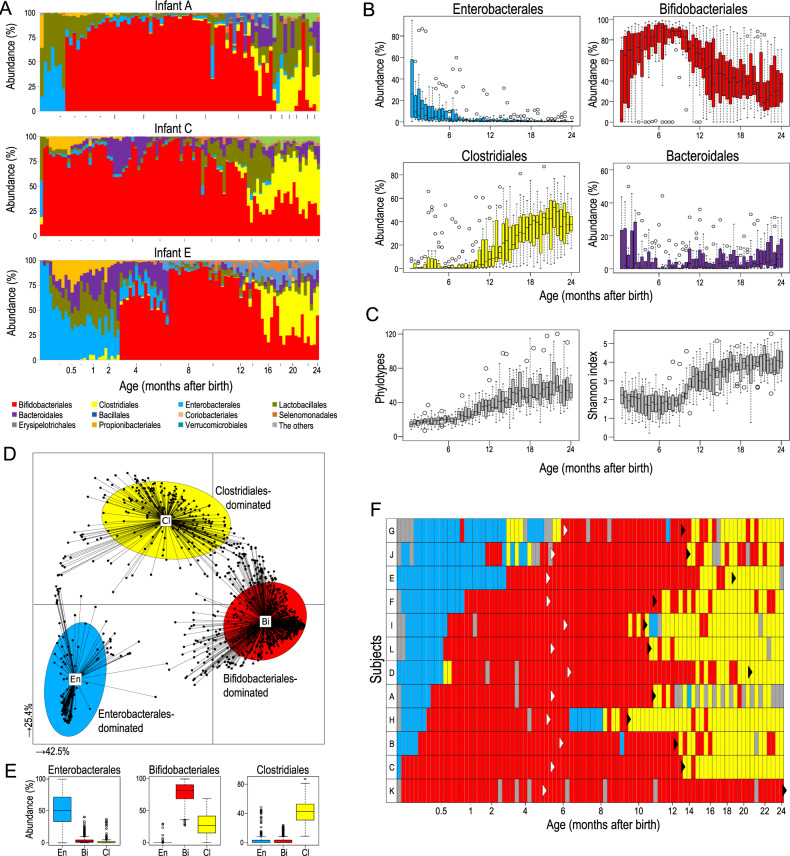
Infant gut microbiota community profiles during the first 2 years of life. Microbiota profiles of faecal samples from 12 infants (*n* = 1048; up to 92 samples per subject) were investigated. **A** Order-level dynamics of three infants (see Fig. S2A for all subjects). Vertical bars along the *x*-axis indicate every 2 months; dots along the *x*-axis indicate every week until 1 month. **B** Temporal shift of bacterial abundance in 12 infants at the order level. **C** Temporal shift in α-diversity. **D** Characteristics of infant gut microbiota, illustrated using JSD with PCoA and PAM clustering analyses. **E** Box plots showing relative abundances of the main contributors to each cluster. **F** Temporal shift of microbiota clusters. Light blue, Enterobacterales dominant; red, Bifidobacteriales dominant; yellow, Clostridiales dominant; grey, not tested. White and black arrowheads indicate the initiation of solid food and cessation of breastfeeding, respectively. Subjects were ordered with a stable colonisation of Bifidobacteriales.

We then sequenced the amplicons of the V1 and V2 regions of 16S rRNA genes of these infant faecal samples, along with specimens obtained from their parents (a total of 1070 samples). A total of 24 million amplicon sequences (average: 22,956 ± 17,959 reads per sample) were analysed. The 16S rRNA-gene amplicon analysis allows characterisation at different phylogenetic levels, and the results at level 4 (order in taxonomic hierarchy) are presented in Fig. 1 (see Fig. S1 for different taxonomic levels).

The early microbiome showed considerable variation in both composition and progression among individuals (An age-dependent gut microbiota composition of three infants are shown in Fig. 1A. See Fig. S2A for all 12 infants). However, they also exhibited a temporal structure (Fig. 1B). Initial microbiota was typically dominated by Enterobacterales, but their abundance decreased with age. Bifidobacteriales was the major bacterial order in early life microbiota, showing increasing average abundance up to 6 months of age and a general decline after 8 months. Clostridiales were the minor bacterial lineages until 8 months of age, but their abundance increased thereafter.

We also confirmed that α-diversity, as evaluated by the number of phylotypes, Shannon index and Faith’s phylogenetic diversity (PD), increased with age (Figs. 1C and S2B). At the end of the test period, the increase in these diversity indexes slowed down, although the values in these infants at 2 years of age were still significantly lower than those of their parents (Fig. S2C).

Characteristics of the microbiota in these samples were then evaluated using Jensen–Shannon divergence (JSD) with principal coordinate analysis (PCoA) and partitioning around medoids (PAM) clustering algorithm (Fig. 1D). We used Calinski–Harabasz (CH) index for estimating the number of clusters [30] and found the infant microbiota to be divided into three clusters. The infant microbiota-specific clusters, denoted as microbiota clusters En, Bi and Cl, were characterised by the predominance of Enterobacterales, Bifidobacteriales and Clostridiales (Fig. 1E).

We then visualised the clusters belonging to each sample with respect to age and found a progression from Enterobacterales- to Bifidobacteriales- and then to Clostridiales-dominant microbiota (Fig. 1F). The day of transition exhibited significant inter-individual variation; the transition from Enterobacterales to Bifidobacteriales ranged from 3 days to 6 months (median, 0.6 month), whereas that from Bifidobacteriales to Clostridiales ranged from 8 to 24 months (median, 13 months). Consistent with previous studies [14, 31], the transition from Bifidobacteriales- to Clostridiales-dominant microbiota coincided with breastfeeding cessation in many infant subjects (Fig. 1F, black arrowhead).

### Dynamics and individuality of SCFA profiles in early life

To gain an understanding of the pattern and inter-individual variation of the early life gut SCFA profile, we analysed the concentration of SCFAs in these faecal samples using high-performance liquid chromatography (HPLC).

**Fig. 2:**
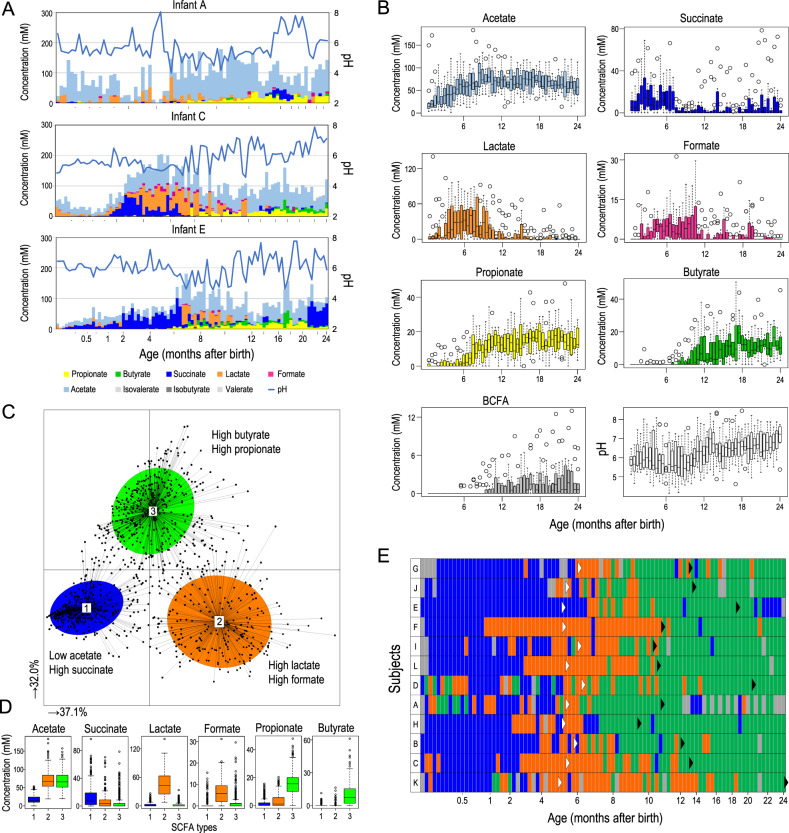
Infant gut SCFA profiles during the first 2 years. **A** Faecal SCFA and pH dynamics of three infants (see Fig. S3A for all subjects). **B** Temporal shift of faecal SCFA concentrations and pH. **C** Characteristics of infant gut SCFA profiles, illustrated using JSD with PCoA and PAM clustering analyses. **D** Box plots showing faecal SCFA concentration of the main contributors to each cluster. **E** Temporal shift of the SCFA clusters. Blue, acetate declined and succinate elevated (type 1); orange, lactate and formate elevated (type 2); green, propionate and butyrate elevated (type 3); grey, not tested. White and black arrowheads indicate the initiation of solid food and cessation of breastfeeding, respectively. The subjects were ordered as in Fig. 1F.

Similar to those in the development of gut microbiota, the profiles of gut SCFAs in early life were dynamic and individualised (Figs. 2A and S3A and Data File S1), whereas the patterns showed temporal trajectories (Fig. 2B). Acetate was the primary SCFA throughout the test period; its concentration increased toward 6 months of age and was stable thereafter (Fig. 2B). The concentration of succinate, lactate and formate was elevated in early life but declined until 1 year of age. The other major SCFAs, propionate and butyrate, increased after 8 and 10 months of age, respectively. Branched-chain fatty acids (i.e., isobutyrate and isovalerate) were rarely detected throughout the test period (especially until 9 months of age). We also observed increasing faecal pH during the first 2 years; the median pH value was lower than 6.0 until 10 months of age and higher than 6.0 thereafter (Fig. 2B).

We subsequently tried to characterise the gut metabolite profiles using JSD with PCoA and PAM clustering, as we have done for microbiota characterisation (Fig. 2C). Based on the CH index, we found the SCFA pattern to be clustered into three types, characterised by either low acetate and high succinate concentrations (SCFA type 1), high lactate and high formate concentrations (SCFA type 2) or high propionate and high butyrate concentrations (SCFA type 3) (Fig. 2D).

We then summarised the progression of the SCFA type and observed sequential transitions from SCFA types 1 to 2 and then to type 3, with individual variation on the day of transition (Fig. 2E). The transition from types 2 to 3 SCFA profile, driven by the elevation of propionate concentration, was observed prior to breastfeeding cessation, whereas the increase in butyrate concentration and reduction of lactate and formate coincided with the termination of breastfeeding (Fig. S3B).

### Association between gut SCFA profiles and microbiota

**Fig. 3:**
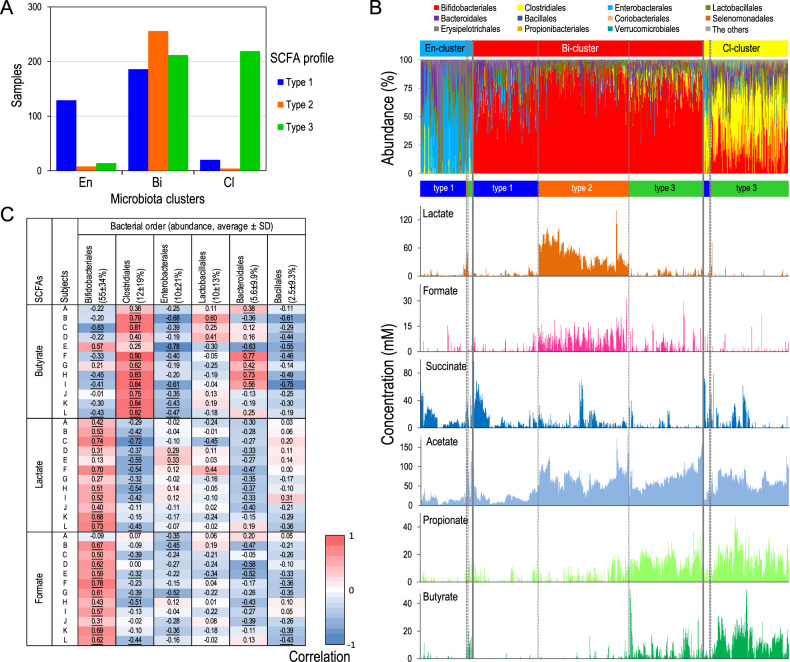
The relationship between infant microbiota composition and gut SCFA profiles. **A** Relationship between three microbiota clusters and three SCFA patterns. **B** Microbiota composition (ordered from cluster En, Bi to Cl) and SCFA profiles (ordered from types 1 to 3) are shown. **C** Personalised association between gut SCFA profile and microbiota at the order- level (see Fig. S4 for the other SCFAs). Association with the top 6 bacterial lineages (average abundance > 2%) is presented. Numbers represent *r* values (Spearman’s correlation). Underlined numbers indicate FDR-corrected *p* values < 0.01.

To consider the relationship between gut SCFA and microbial community structure, we subsequently evaluated the association between microbiota clusters and SCFA types (Fig. 3). We found that most samples with Enterobacterales-dominant microbiota exhibited the type 1 SCFA profile with reduced acetate and increased succinate (129 of 151, 85.4%) and that most Clostridiales-dominant microbiota showed the type 3 SCFA profile with increased propionate and butyrate (219 of 243, 90.1%) (Fig. 3A). Noteworthy, Bifidobacteriales-dominant microbiota (*n* = 654) exhibited diverse SCFA types; 186 samples showed the type 1 SCFA profile (28.4%), 256 samples showed the type 2 SCFA profile with increased lactate and formate (39.1%) and 212 samples showed the type 3 SCFA profile (32.4%). A direct comparison of gut microbiota compositions and SCFA concentrations is shown in Fig. 3B, emphasising that the different concentrations of SCFA (including lactate and formate) were observed among Bifidobacteriales-dominant microbiota.

A dense longitudinal data set has been demonstrated to be adequate to assess the association between microbiota composition and environmental factors [32]. Therefore, we calculated Spearman’s correlation coefficient between the concentration of SCFAs and bacterial taxonomic profiles in each infant, according to the method of Johnson [32], to identify bacterial lineages that play major roles in each SCFA production (Figs. 3C and S4). Although the association between SCFAs and microbiota showed variation among individuals, the highest average correlation coefficient was observed between Clostridiales and butyrate (*r* = 0.68 ± 0.21), and was significant in 11 infants (*q* < 0.01, *p* value adjusted with FDR). We also found positive association between Clostridiales and propionate (*r* = 0.63 ± 0.23), Bifidobacteriales and formate (*r* = 0.50 ± 0.19), and Bifidobacteriales and lactate (*r* = 0.52 ± 0.22) at bacterial order-level evaluations, which are further discussed below.

### Contribution of Clostridiales to gut butyrate production

**Fig. 4:**
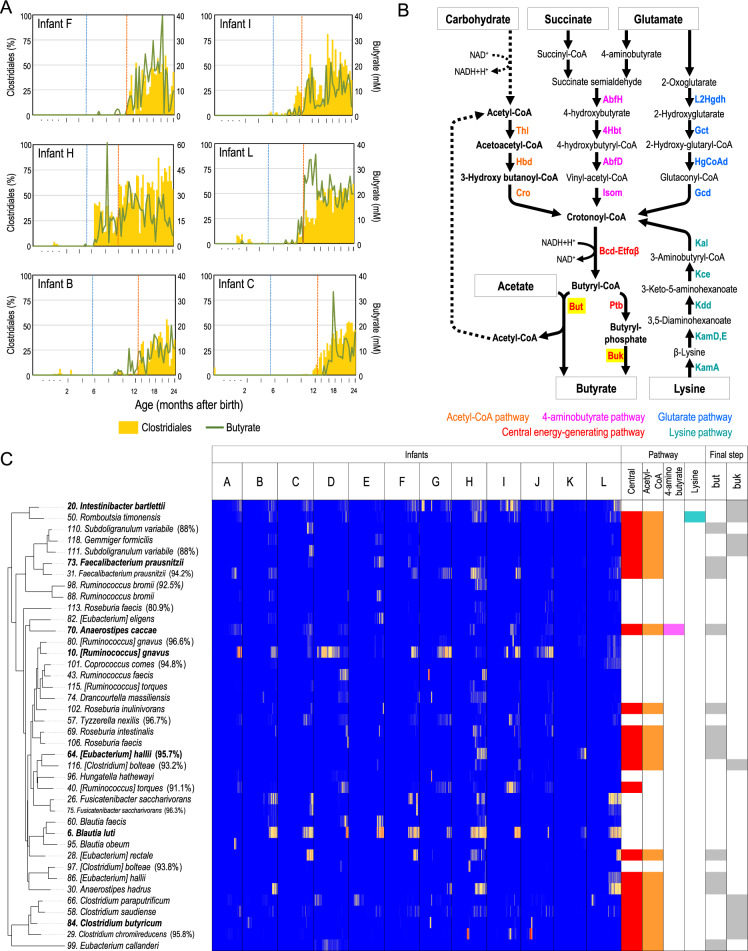
Clostridiales members contribute to gut butyrate production. **A** Plot of Clostridiales abundance and butyrate concentrations with respect to age. Six representative subjects are presented (see Fig. S5A for all subjects). Blue and orange dotted vertical lines represent the initiation of solid food and cessation of breastfeeding, respectively. **B** Pathway for butyrate production [35]. **C** Age-dependent heatmap of Clostridiales phylotypes and their potent butyrate production. The top 40 Clostridiales phylotypes are shown. Left tree represents the taxonomic relationship based on 16S rRNA sequences. Right figure is a summary of the presence of genes for butyrate production (see Fig. S6B). Phylotypes mentioned in the text are highlighted in bold.

Because a prominent positive association was observed between Clostridiales and butyrate, we evaluated their relationship by visualising the abundance of Clostridiales and concentration of butyrate with respect to age. As shown in Figs. 4A and S5A, we found that the increase in gut butyrate concentration coincided with elevation of Clostridiales abundance. The results are consistent with previous reports showing that some gut microbes belonging to Clostridiales (e.g., *Eubacterium rectale*, *Roseburia intestinalis* and *Faecalibacterium prausnitzii*) play roles in gut butyrate production [33, 34].

On the other hand, recent studies reported that not all Clostridiales phylotypes contribute to gut butyrate production [33, 34], which prompted us to investigate the Clostridiales phylotypes and genes involved in gut butyrate production. In this study, 370 phylotypes belonging to Clostridiales were detected in 12 infants during the first 2 years of life; the distribution and progression of the top 80 abundant phylotypes are visualised in a heatmap (Fig. S6A). We observed that *Blautia luti*, *Ruminococcus gnavus* and *Intestinibacter bartlettii* were detected frequently, although they were not always dominant in all the subjects.

Genome sequences of Clostridiales species have been accumulating in public databases, and the study by Vital et al. [35] detailed the genes associated with four major pathways that produce butyrate (Fig. 4B). Therefore, we investigated whether the phylotypes detected in the present study possessed the genes for butyrate production (see “Materials and Methods” for details). Consistent with previous studies, we found that only a part of Clostridiales phylotypes (45 out of top 80) possessed the pathway (Fig. S6B). The majority of butyrate-producing Clostridiales (21 out of top 80 phylotypes including *F. prausnitzii*, *E. rectale* and *Eubacterium hallii*) possessed the genes for acetyl-CoA pathway with butyryl-CoA:acetate CoA transferase (*but*) (Figs. 4C and S6B), suggesting that microbiota-accessible carbohydrates and gut acetate produced by other gut microbes are the substrates of gut butyrate production. Some species (18 out of 80 phylotypes including *Clostridium butyricum*) possessed butyrate kinase (*buk*), instead of *but*, as the enzyme that catalyses the final step from butyryl-CoA to butyrate (Figs. 4C and S6B); the results indicate that these species uses carbohydrates, but not acetate, for butyrate production. Some species possessed the 4-aminobutyrate/succinate pathway (12 phylotypes including *Anaerostipes caccae*), suggesting that succinate (produced by other gut microbes) could also be used for butyrate production.

Figure S5B shows the plot of butyrate concentration and the sum of Clostridiales phylotypes possessing butyrate-producing pathways with respect to age, confirming that the increase in butyrate-producing Clostridiales and butyrate concentration is consistent in many subjects.

Thus, our study identified that diverse and personalised assemblage of Clostridiales phylotype that possess the acetyl-CoA pathway play major roles in gut butyrate production (Fig. 4C) and that not all Clostridiales phylotype contribute to this metabolism. We also detailed the species and genes involved in butyrate production, by combining 16S rRNA amplicons and gene catalogues for butyrate metabolism [35] (Supplementary Text), enabling a discussion on the substrates for gut butyrate production in early life.

### Only some infant-type Bifidobacteriales correlate with gut SCFAs

**Fig. 5:**
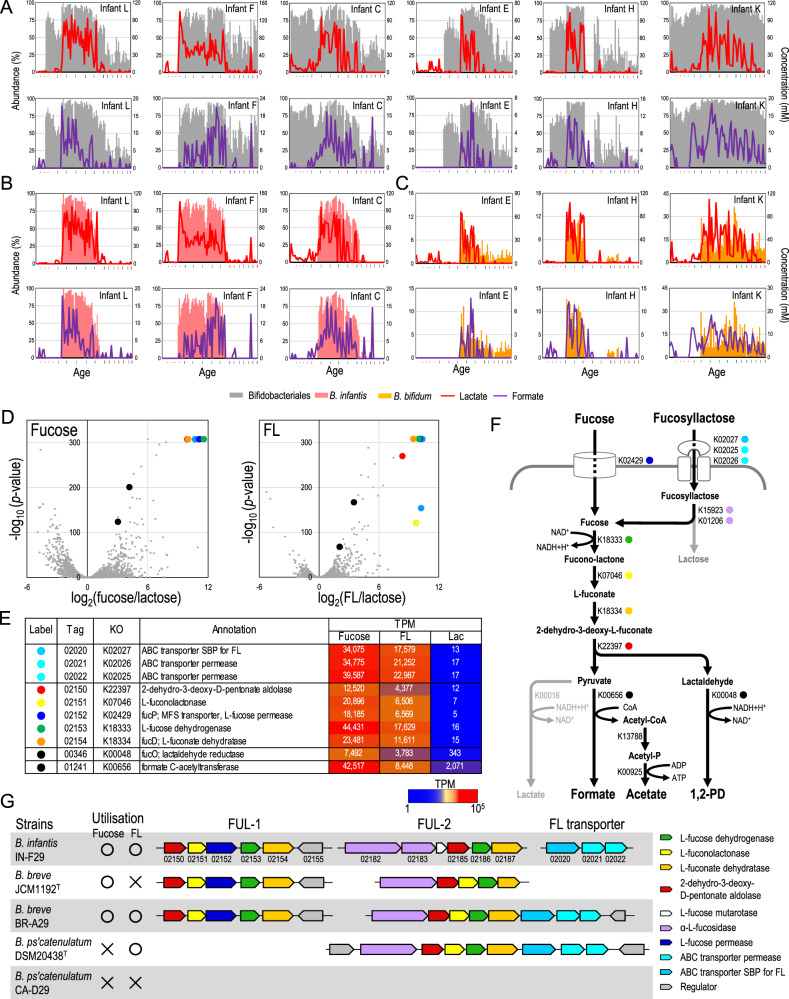
Infant bifidobacteria contribute gut lactate and formate production. **A**–**C** *Bifidobacterium* abundance and concentration of lactate and formate with respect to age. Six infants whose lactate and formate production were driven by *B. infantis* or *B. bifidum* are presented (see Fig. S7 for all subjects). **D** Volcano plots of transcriptional data during utilisation of fucose and FL compared to lactose. **E** List of upregulated genes and their locus tags (INF29_xxxxx are abbreviated), KEGG orthology (KO), annotation and transcripts per million (TPM). **F** Predicted metabolic pathway from fucosyllactose and fucose to formate. **G** Organisation of fucose utilisation genes and their phenotypes (see Fig. S10A,B for the other strains). Numbers below the arrow represent the last digits of the locus tags.

Subsequently, we evaluated the relationship between the abundance of Bifidobacteriales and concentrations of lactate and formate by plotting these values with respect to age. However, unlike the Clostridiales–butyrate relationship, the increase in Bifidobacteriales abundance and concentration of these two SCFAs did not coincide; increase in Bifidobacteriales was observed prior to the increase in lactate and formate in most subjects (Figs. 5A and S7A).

Therefore, we evaluated the Bifidobacteriales-SCFA association at the species level (Fig. S8). Spearman’s correlation coefficient within each subject indicated that *Bifidobacterium longum* subsp. *infantis* (denoted as *B. infantis* thereafter), *Bifidobacterium bifidum* and *Bifidobacterium breve* were positively associated with gut lactate and formate concentrations, and that the bifidobacterial species associated with these SCFAs varied among individuals (Fig. S8). We also evaluated their relationship by plotting the abundance of these species and the SCFA concentrations with respect to age (Figs. 5B, C and S7B). We found that the increase in *B. infantis* abundance coincided with increased lactate and formate concentrations in infants with positive correlation (e.g., subjects L, F and C; Fig. 5B), whereas increase in *B. bifidum* abundance and gut SCFA concentrations coincided in the other infants (e.g., subjects E, H and K; Fig. 5C). These data together suggested that some, though not all, bifidobacterial species contribute to the production of lactate and formate in the infant gut, and have the same functional roles in the ecosystem, which is referred to as functional redundancy.

### *B. infantis* and *B. breve* assimilate HMO-derived fucose and produce formate

*B. infantis*, *B. bifidum* and *B. breve* are commonly found in the infant gut, and the former two species possess an arsenal of genes for utilising HMOs [4, 36, 37]. The HMOs, known as major microbiota-accessible carbohydrates in early life, contain glucose and galactose as the main components, with fucose, *N*-acetylglucosamine and *N*-acetylneuraminic acid residues as sub-components. Bifidobacteria metabolise galactose to glucose via the Leloir pathway, and glucose is metabolised into acetate and lactate at a 3:2 ratio via a bifidobacteria-specific “bifid shunt” pathway (Fig. S9A) [38]. Therefore, increased gut lactate concentration, coupled with *B. infantis* and *B. bifidum*, were attributed to their ability to utilise HMO. This presumption is consistent with the observation that increase in lactate coupled with these bifidobacterial species abundances coincided with increased acetate concentration in many of these infants (Fig. S7B, indicated by arrows).

However, bifidobacterial metabolic pathways to produce formate and its substrate in the infant gut have not been sufficiently explored. Therefore, we investigated the utilisation and metabolic consequences of HMO-derived monosaccharides (fucose, *N*-acetylglucosamine and sialic acid) using *B. infantis* strain IN-F29 and found that the strain produced formate through the fermentation of fucose.

We subsequently performed RNA-seq transcriptional analysis of the *B. infantis* strain during its growth in presence of fucose, 2′-fucosyllactose (FL), or lactose, to investigate the molecular basis underlying the metabolism of HMO-derived fucose into formate. We found that eight genes were significantly upregulated during fucose and FL utilisation, many of which have been associated with fucose metabolisms and FL transportation (Fig. 5D, E). We also observed that the genes for the final step for formate (formate C-acetyltransferase [K00656], INF29_01241) and 1,2-propanediol (1,2-PD) production (lactaldehyde reductase [K00048], INF29_00346) were upregulated. Based on the transcriptome data analysis, gene annotation and previous studies [38], we hypothesised that bifidobacteria utilise HMO-derived fucose and produce formate via the pathway shown in Fig. 5F.

The upregulated genes associated with fucose to pyruvate and lactaldehyde metabolism are located in a locus (locus tag INF29_02150–02155), denoted as fucose utilising locus-1 (Fig. 5G). In addition, we found that the other set of genes has the same functional annotations and is located in another locus (Fig. 5G; INF29_02182-02187, denoted as FUL-2). The transcriptional RNA-seq data confirmed that the genes located in FUL-2 were constitutively expressed during fucose, FL and lactose utilisation (Fig. S9B), suggesting that these genes are also involved in HMO-derived fucose utilisation.

We further investigated the presence of these genes (for HMO-derived fucose utilisation) in 39 representative *Bifidobacterium* strains, and found that all strains of *B. infantis* and *B. breve* possessed the genes for the metabolic pathway from fucose to formate (Fig. S10A, B). The ABC transporter for FL (K02025–02027) was not found in some *B. breve* strains (e.g., JCM1192^T^, BR-07, BR-19, BR-C29, BR-H29 and BR-L29), which is consistent with the observation that these strains are unable to utilise FL [4]. In contrast, we observed that some *B. pseudocatenulatum* strains possess FUL-2 along with all the genes for the conversion of fucose to formate and 1,2-PD. These strains contained the genes for ABC transporter for FL in the locus but the fucose permease gene was absent, which is consistent with their phenotype of being capable of utilising FL but not fucose [4]. Thus, the variation in FL and fucose utilisation observed among infant-type bifidobacteria can be explained by the presence of genes for FL and fucose transporters and their related pathways, as summarised in Figs. 5G and S10C–G.

### *B. bifidum* contributes to gut formate production by cross-feeding of fucose

**Fig. 6:**
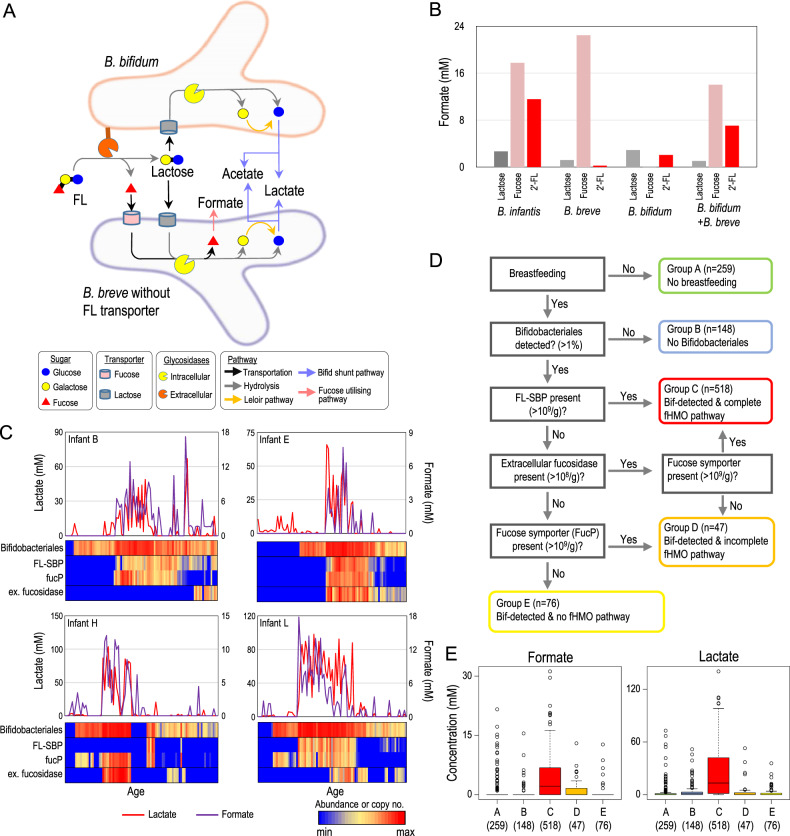
Fucosylated HMO utilisation and production of formate by the bifidobacterial community. **A** Proposed cross-feeding model for FL metabolism by *B. bifidum* and *B. breve* to produce SCFA. **B** Formate production by *B. infantis*, *B. breve*, *B. bifidum* and a combination of these species. **C** Relationship between key marker genes for fucosylated HMO utilisation and concentration of lactate and formate (see Fig. S11 for all subjects). **D** Samples were divided into five subgroups based on feeding, Bifidobacteriales-colonisation and presence of key genes for fucosylated HMO utilisation. **E** Difference in faecal SCFA concentration among the subgroups. Digits in parenthesis represent the number of samples assigned to the subgroup.

Interestingly, but consistent with previous studies [5], *B. bifidum* lacks the metabolic pathway to assimilate fucose (Figs. S10A), although its abundance was positively associated with gut formate concentration in several infants (Figs. 5C, S7 and S8B). Previous in vitro studies have reported that *B. bifidum* possess extracellular fucosidase to utilise fucosylated HMOs [37], thereby releasing fucose into the culture supernatant used by *B. breve* for further metabolism [5, 39]. However, the metabolic consequences of the co-culturing of these two *Bifidobacterium* species have not been investigated. In the present study, *B. breve* was detected in most samples in which the formate–*B. bifidum* association was observed (Fig. S7). Therefore, we assumed the increase in formate coupled with *B. bifidum* colonisation to the substrate cross-feeding of fucose by *B. bifidum* and *B. breve* (Fig. 6A).

To test this hypothesis in vitro, we cultured the representative strains of *B. infantis*, *B. bifidum*, and *B. breve* (with no ABC transporter for FL) in media containing fucose, 2′-FL, and lactose (see Supplementary Text for details). We confirmed that *B. infantis* produced formate using both fucose and 2′-FL; *B. breve* produced formate with fucose but not with 2′-FL; and *B. bifidum* produced less formate with fucose nor with 2′-FL (Fig. 6B and Table S2). In contrast, the co-culture of *B. breve* and *B. bifidum* utilised 2′-FL and produced formate, confirming our hypothesis that a combination of these two species could utilise fucosylated HMO to produce formate by cross-feeding.

### In vivo fucosylated HMO utilisation and formate production

To evaluate the notion that both bifidobacteria with ABC transporter for FL and the fucose cross-feeding contribute to formate production in our infant data, we further investigated the presence of these key genetic factors and their association with the gut SCFA concentration.

We developed specific primers for substrate-binding protein for FL (denoted as FL-SBP) [4], fucose symporter (FucP) and extracellular fucosidase and investigated their presence in 1048 samples obtained from the 12 infants using real-time PCR (Figs. 6C and S11). As expected, the increase in the FL-SBP gene, which is always present with FUL, corresponded with an increase in gut formate and lactate concentration in many subjects (e.g., infants B, C, K and L). Although the correlation was not observed in several infants, the increase in gut formate and lactate concentration could be explained by the co-presence of extracellular fucosidase and FucP, both of which are key genetic factors for fucose cross-feeding (e.g., infants H and E).

We further divided these 1048 samples into five subgroups based on feeding, the colonisation of Bifidobacteriales and the presence of the key genes for fucosylated HMO utilisation (see Fig. 6D). We confirmed that the samples with a complete set of fucosylated HMO-utilising pathway showed a higher formate and lactate concentration than the samples in the other subgroups (Fig. 6E), supporting our concept that both the ABC transporter for FL and the bifidobacterial cross-feeding of fucose contribute to formate production in the infant gut.

## Discussion

Gut microbiota-derived SCFA have been shown to exert a wide range of physiological effects on the host [17], whereas gut microbes have different capacities to produce and metabolise these SCFAs [16, 35]. Therefore, the microbiota with different compositions result in different gut SCFA profiles, which may exert distinct host responses. However, the detailed relationships with gut microbiota have been poorly delineated [40]. In this study, we characterised the gut SCFA profiles in relation to gut microbiota composition during the first 2 years using dense longitudinal sampling. We found the gut SCFA profiles to exhibit three phases of progression. We further investigated the key bacterial lineages, genetic factors and life events that affect gut SCFA profiles in early life. Thus, the data presented in this study improve our understanding of the early life gut SCFA profile and its relationship with gut microbiota.

### Lactate and formate production by HMO-utilising bifidobacteria

In this study, we demonstrated lactate and formate to be the major metabolites in the 2nd phase of the SCFA profile. Although the duration of increased lactate and formate steady-state differed among subjects (1–10 months, Fig. 2E), lactate- and formate-elevated phases were observed in all subjects in this study. Previous studies in adults have reported that these two SCFAs do not accumulate in the gut because they are intermediates and are converted to other metabolites [17, 41]. Some infant studies did explore faecal SCFA concentration [11, 42]; however, they only focused on gut acetate, propionate and butyrate, whereas gut lactate and formate concentrations have not been focused in most studies. Thus, to our knowledge, this is the first report to emphasise that formate and lactate accumulate in the gut for a certain period in healthy infants.

Furthermore, we demonstrated that fucosylated HMO-utilising bifidobacteria play key roles in gut SCFA production. We focused on formate production and found that HMO-derived fucose is the major substrate for producing the SCFA in the infant gut, and we propose a detailed bifidobacterial metabolic pathway from fucose to formate. The proposed pathway is consistent with recent publications reporting that some *Bifidobacterium* strains (e.g., *Bifidobacterium kashiwanohense* strain APCKJ1) metabolise fucose to 1,2-PD [43, 44]. Our study corroborates their findings and further showed that the gene INF29_01241, annotated as formate C-acetyltransferase, is involved in the final step to produce formate and complement the overall picture of fucose metabolism in the infant gut symbiont bifidobacteria.

Regarding the metabolism of fucose and lactose, it is worth noting that fucose utilising bifidobacteria possess the enzyme to convert pyruvate not only to formate but also to lactate. We observed that *B. infantis* and *B. breve* produced formate, acetate and 1,2-PD thorough fucose fermentation, and that they produced acetate and lactate through lactose metabolism (Table S2). We also confirmed that genes associated with pyruvate-to-formate conversion (K00048, K00656 and K13788) were upregulated in fucose utilisation, genes for pyruvate-to-lactate conversion (K00016) were upregulated in lactose utilisation and genes involved in both (K00925) were constitutively expressed in *B. infantis* (Fig. S9C). This regulation can be explained by the NADH/NAD^+^ balance in the fucose and lactose metabolic pathways. NAD^+^ is used for fucose to fucono-lactone conversion and regenerated during lactaldehyde to 1,2-PD conversion (Fig. S9A), and the pyruvate can be further metabolised to acetate coupled with carboxylation of CoA by formate C-acetyltransferase. However, pyruvate cannot be further metabolised efficiently to lactate during fucose utilisation, as the NADH/NAD+ is balanced without this reaction.

The physiological effects of microbiota-derived formate and lactate have been less actively investigated, probably owing to its lower concentration in the gut lumen under normal physiological conditions in adults. The present study demonstrated the production and accumulation of lactate and formate in breastfed infants, thereby highlighting the importance of investigating the functional roles of these minor SCFAs.

### Butyrate production by diverse and personalised Clostridiales phylotype

In addition to previous findings regarding the increase in several groups of Firmicutes (including Clostridiales) coinciding with cessation of breastfeeding [1, 14, 31], our present study highlights the advanced notion that the cessation induces the elevation of gut butyrate production (Figs. 2E, S3 and S5). We also confirmed that introduction of solid food had little impact on the SCFA profile, as well as on gut microbiota composition [14, 31].

These observations suggest the presence of specific factors that control gut butyrate production in human milk. Human milk has long been recognised to contain large amounts of lactoferrin [45]. Lactoferrin limits the availability of free Fe in the environment, which is essential for bacteria to perform enzymatic reactions and regulate gene expression [46]. Previous studies demonstrated that bifidobacterial species are well adapted to low-Fe conditions [47] and that Fe-deficiency or chelation results in decreased butyrate production both in in vitro colonic fermentation and in animal models [48, 49]. In this study, we found that the acetyl-CoA pathway is the major pathway in butyrate-producing Clostridiales in early life. The central energy-generating step of this pathway is the transformation of crotonyl-CoA to butyryl-CoA (Fig. 4B), which creates a proton motive force via ferredoxin reduction by the butyryl-CoA dehydrogenase electron-transfer flavoprotein complex [35, 50]. These previous findings and our observation lead us to consider that cessation of breastfeeding may result in decreased lactoferrin and increased free Fe, thereby leading to increased gut butyrate production: this hypothesis should be further validated in future investigations.

### Other associations among gut SCFA, microbiota and environmental factors

We observed a positive correlation between Clostridiales abundance and propionate concentration (Fig. S4). However, the increase in propionate was observed prior to the elevation of Clostridiales abundance (Fig. S3), and thus we were not able to propose convincing causal relationship in this study.

Consistent with our observation that most Enterobacterales-dominant microbiota exhibited the type 1 SCFA profile with increased succinate (Fig. 3a), Bittinger et al. [51] recently reported that *Escherichia coli*, a representative species belonging to Enterobacterales, contribute succinate production through amino acids metabolism, based on the observation of metabolite in newborn faeces and a nutrient flux balance model analysis. The other data in this study (Fig. S12) also support their finding since the elevation of Enterobacterales abundance and succinate concentration with respect to age was correlated in some infant subjects (e.g., subject F, G and I, Supplementary Text [52]). In this way, our present SCFA–microbiota data set can be used to support or evaluate the detailed gut SCFA–microbiota relationship and may provide a valuable resource for future investigation.

Exposure to antibiotics in early life has been associated with increased risk of developing allergies and asthma, being overweight and enhanced adiposity [2]. Our study provides case-control data on how antibiotic treatment affects gut SCFA profiles and the microbiota. In this study, 8 out of 12 subjects received antibiotics during the first 2 years, many of which showed alterations in SCFA profile, microbiota composition and/or α-diversity (Figs. S2A, B and S3A, indicated by arrowheads). In addition, we observed that these changes were reverted to their pre-treatment state after completion of the treatment, although the magnitude of the effects and its recovery varied among individuals.

In this study, we observed a negative correlation between gut pH and lactate concentration (*r* = −0.58 ± 0.19, Fig. S13). This observation and our in vitro data suggested the involvement of HMO-utilising bifidobacteria to produce the SCFA, thereby contributing to lowering gut pH. Conversely, environmental pH has been shown to affect the composition of the microbiota [53]. Some studies proposed that low pH could be a critical factor to inhibit the overgrowth of potential pathogens in the gut ecosystem [54]. The ability of bifidobacteria to tolerate acidic environments has also been actively investigated [55]. Thus, our data and the previous findings implicate a positive feedback loop between gut pH and a Bifidobacteriales-dominated microbiota.

### Infant health and microbiota-derived SCFA

Infant gut microbiota development is now being actively investigated in relation to health and disease risk of the host later in life [9, 10, 13]. In this study, we identified three distinct SCFA patterns that showed sequential transition, which exhibited considerable individual variation; however, the association between the progression of SCFA profiles, environmental factors (other than breastfeeding cessation) and subsequent infant health could not be adequately evaluated, at least in part owing to the small number of subjects. Therefore, it will be interesting to extend future infant birth cohorts to target both microbiota and SCFA profiles with frequent sampling to better understand the gut microbiota–SCFA–environment–host health relationship.

Knowledge of the developmental pattern of gut SCFA and its association with microbiota detailed in this study is an important step for developing a strategy to modulate gut SCFA in early life, which may affect lifelong host health.

## Materials and methods

### Subject recruitment and sample collection

A total of 1070 faecal samples were investigated as an extension of a previously described infant study [4]. The study was conducted according to the guidelines in the Declaration of Helsinki, approved by the ethical committee of Yakult Central Institute and written informed consent was obtained from the parents before enrolment. All infants were of Japanese origin, term, mainly breastfed and born vaginally (Table S1). Parents were instructed to record changes in diet, medications, hospitalisation, birth weight and gestational age. The samples were frozen at −20 °C, transferred to the laboratory and stored at −80 °C.

### 16S rRNA-amplicon analysis

DNA was extracted using bead-beating in phenol, as described previously [4]. Variable regions 1 and 2 (V1 and V2) of the 16S rRNA gene were amplified from faecal DNA using the primers 27Fmod2-MiSeq (5′-AATGATACGGCGACCACCGAGATCTACACTCTTTCCCTACACGACGCTC-TTCCGATCT-AGRGTTYGATYMTGGCTCAG-3′) and 338RMiSeq (5′-CAAGCAGAAGACGGCATACGAGAT-NNNNNNNNNN-GTGACTGGAGTTCAGACGTGTGCTCTTCCGATCT-GCTGCCWCCCGTAGGW GT-3′). The amplicons (250 bp, paired-end) were sequenced using an Illumina MiSeq platform (MiSeq reagent kit v2).

### Amplicon-based microbiota analysis

Sequences generated from the Illumina MiSeq were analysed using the QIIME2 software package (version 2018.8) [56]. DADA2 algorithm was used to remove low-quality reads, de-noise, concatenate the 16S rRNA reads and remove potential chimeric sequences [57]. The resultant features were subsequently clustered into phylotypes (clustered feature) using q2-vsearch for open-reference clustering against the NCBI 16S RefSeq records (downloaded on December 12, 2019) [58].

The taxonomy of each phylotype was assigned using the qiime feature-classifier [59] against the SILVA database (Release 138) [60] with a minimum bootstrap threshold of 50%. A single representative from each phylotype was aligned using the MAFFT alignment tool [61], and a phylogenetic tree was constructed using FastTree [62]. α-Diversities (the number of phylotypes observed, Shannon index and Faith’s PD) were estimated for 5000 randomly selected sequences to account for the differences in sampling efforts among the samples. The phylogenetic relationship of the target phylotypes was analysed with their 16S rRNA-gene sequence (V1 and V2 regions) using MAFFT [61] with default settings, and the phylogenetic tree was visualised using FigTree (http://tree.bio.ed.ac.uk/software/figtree/). For heatmap visualisation, the colour scales function of Excel 2013 (Microsoft) was used.

PCoA and between-class analysis were performed according to the procedure described by Arumugam et al. [30]. Data generated by QIIME at the level of bacterial order were used to calculate the Jensen–Shannon divergence (JSD) among samples. The partitioning around medoids (PAM) clustering algorithm was applied to cluster the profiles. The number of clusters was estimated by calculating the CH index according to a previously described method [30]. PCoA and between-class analysis were performed according to the ref. [30].

### Discrimination between *B. longum* subsp. *longum* and subsp. *infantis*

We enumerated *B. longum* subsp. *longum* and subsp. *infantis* separately, although 16S rRNA amplicon-based analysis targeting V1 and V2 regions did not allow accurate assignment at the subspecies level for this species. We used specific PCR primers to detect *B. longum* subsp. *longum* (BiLON-1; 5′-TTCCAGTTGATCGCATGGTC-3′ and BiLON-2; 5′- GGGAAGCCGTATCTCTACGA-3′) [63], and *B. longum* subsp. *infantis* (Binf1-F: 5′-AGCAGCAGAAGTCCAGTGAAG-3′ and Binf1-R: 5′- AGTAGTGGATGGTCGGCATAC-3′) (Supplementary Table 3). To design the specific primers for *B. longum* subsp. *infantis*, all the genome sequences belonging to human bifidobacteria (i.e., *B. longum* subsp. *longum*, *B. longum* subsp. *infantis*, *B. breve*, *B. catenulatum*, *B. pseudocatenulatum, B. adolescentis* and *B. bifidum*) were obtained from RefSeq database (on March 2019), and we found that strains belonging to *B. infantis* specifically contain the sodium/glucose cotransporter gene (GCF_000269965.1_ASM26996v1_genomic_01458). The primers were then designed using Primer3Plus [64] and evaluated with Primer-BLAST [65] with default setting. Real-time PCR was performed as described previously [63]. The abundance of *B. longum* subsp. *infantis* and *B. longum* subsp. *longum* was estimated by integrating the sum of 16S rRNA amplicons of these subspecies and ratio of the enumeration by qPCR, for each subspecies.

### Determination of pH, SCFAs and carbohydrate concentration

Faecal pH was determined in triplicate with a hand-held pH metre (model IQ150) equipped with a PH17 SS electrode (IQ Scientific Instruments, San Diego, CA, USA). The SCFA concentrations were determined as previously described [4]. Briefly, the samples (faecal dilution or culture supernatant) were mixed with perchloric acid (2% final concentration), incubated at 4 °C for 3 h and centrifuged at 13,000 × *g* for 5 min at 4 °C. The supernatant was then filtered through Centricut W-MO (MF 0.45 μm; Kurabou, Osaka, Japan) and analysed using a HPLC system equipped with 432 conductivity detector (Waters, Milford, MA, USA) and a RSpak KC-811 column (Showa Denko KK, Tokyo, Japan). L-Fucose, lactose, 2′-FL and 1,2-PD concentrations were determined using Prominence HPLC System (Shimadzu, Japan) with a KS-802 column and an RI detector (Showa Denko KK, Japan) with a KS-802 column and an RI detector (Showa Denko KK, Tokyo, Japan). Characteristics of faecal SCFA profiles were evaluated using JSD with PCoA and PAM clustering algorithm, as described by Arumugam et al. [30].

### Bacterial strains and culture

The strains used in this study were obtained from Yakult Culture Collection (Tokyo, Japan) and Japan Collection of Microorganisms (Ibaraki, Japan). Bifidobacterial strains were routinely cultured at 37 °C in an anaerobic chamber (Coy Laboratory, Grass Lake, MI, USA) with 88% N_2_, 5% CO_2_ and 7% H_2_, using GAM Broth (Nissui Pharma, Japan) supplemented with 0.5% lactose and 0.5% glucose. The carbohydrate profile was evaluated at 37 °C in modified PY medium (100 mM PIPES, pH 6.7, 5 g/L peptone, 5 g/L BBL trypticase peptone, 10 g/L Bacto yeast extract, 8 mg/L CaCl_2_, 19.2 mg/L MgSO_4_·7H_2_O, 40 mg/L K_2_HPO_4_, 40 mg/L KH_2_PO_4_, 0.4 g/L NaHCO_3_, 80 mg/L NaCl, 4.9 mg/L hemin, 0.5 g/L L-cysteine hydrochloride, 100 ng/L vitamin K1 and 0.1% lactose) supplemented with filter-sterilised 2′-FL (Advanced Protein Technologies, Korea), fucose or lactose. Growth was monitored by measuring optical density at 600 nm every 0.5 h in an anaerobic chamber (Coy Laboratory, MI) using a PowerWave 340 plate reader (BioTek, VT, USA).

### Statistical analysis

We used R (v.3.5.1, http://www.R-project.org/) for statistical analysis. To evaluate the association between gut microbiota composition and SCFA concentration, we calculated Spearman’s rank correlation coefficient using the corr.test package. To correct for multiple testing, the Benjamini–Hochberg false-discovery rate-corrected *p* value (*q* value) was estimated using the p.adjust package.

### Estimation of butyrate production from microbiota composition

We used the butyrate gene catalogue by Vital [35] to evaluate the potent butyrate production of Clostridiales phylotype. First, we obtained the draft genome sequences (RefSeq) of Clostridiales (NCBI Taxonomy: ID 186802) from NCBI Genome using TaxonKit (version 0.5.0) and ncbi-genome-download (version 0.2.10)(https://github.com/kblin/ncbi-genome-download). In this study, there were 8796 taxonomic IDs and we obtained a total of 4829 sequences on December 12, 2019. Subsequently, the presence of genes involved in butyrate production was evaluated for the 4829 Clostridiales draft genomes by conducting a BLASTP search (version 2.2.31) using the butyrate gene catalogue [35] with an *e* value cut-off of 10^−5^ and an identity cut-off of 70%. Finally, the phylotypes detected in this study were associated with the top-hit draft genome of Clostridiales by BLASTN search.

### Comparison of fucose utilisation loci among bifidobacterial species

Genome sequences of the strains listed in Fig. S10A, B were obtained from GenBank (http://www.ncbi.nlm.nih.gov/genbank/) or determined as previously reported [4]. Protein-coding sequences (CDSs) were predicted using Prodigal [66]. Functional annotations of bifidobacteria were performed using BlastKOALA [67] against the species_prokaryotes database. To evaluate the presence of ABC transporter-solute-binding protein for fucosyllactose (FL-SBP), we used BLASTP searches with the target gene (FL-SBP, LC068768) [4] using an *e* value cut-off of 10^−6^ and identity cut-off of 50%. Synteny of genes for fucose utilisation was visualised using drawGeneArrows3 (http://www.ige.tohoku.ac.jp/joho/labhome/tool.html). Localisation of the fucosidase proteins was predicted based on their amino-acid sequences using the PSORT web application (http://psort.hgc.jp/form.html).

### Transcriptome analysis of in vitro monoculture

Bacterial cells in the exponential growth phase were collected and subjected for RNA-seq transcriptional analysis as described previously [68]. The reads (an average of 7.7 million reads per sample) were mapped on *B. infantis* IN-F29 genome (Accession No. GCF_000020425.1) using Bowtie 2 (version 2.2.9) [69] with default settings and normalised by transcripts per million. Only protein-coding regions were considered in this study.

## Supplementary information

Supplementary Figs. 1-13 and Tables 1-3

Dataset in Excel format

## Data Availability

16S rRNA-gene amplicon data and bifidobacterial genome sequences are deposited in the DDBJ Sequence Read Archive under BioProject Accession Code PRJDB9469.
