# Supplementary material for: Key bacterial taxa and metabolic pathways affecting gut short-chain fatty acid profiles in early life
Source: ISME J. 2021 Mar 15;15(9):2574–90. doi: 10.1038/s41396-021-00937-7 (PMC8397723; doi:10.1038/s41396-021-00937-7)
Supplement: Supplementary file 1 — Supplementary Figs. 1-13 and Tables 1-3 [file 41396_2021_937_MOESM1_ESM.pdf]

Supplementary Information

Supplementary Text

Prediction of gut butyrate by microbiota composition with bacterial genome

The butyrate gene catalogue used in this study was originally applied for metagenomic data. In this study, we demonstrated the catalogue to also be useful for enumerating the potent butyrate production in combination with 16S rRNA gene-targeted microbiota composition data.

So far, the bioinformatics software package PICRUSt2 has been developed and applied to predict metagenome functional content from 16S rRNA gene information. However, we found PICRUSt2 to have limitations in predicting butyrate production; the prediction of butyrate by PICRUSt2 did not match with the actual butyrate concentration (data not shown). It could, at least in part, be attributed to some important butyrate production-related genes (e.g. butyryl-CoA transferase of *F. prausnitzii*) not being associated with a KO category.

Metabolic consequences of infant bifidobacteria on HMO-derived carbohydrates

We confirmed that *B. infantis* IN-F29, *B. breve* JCM1192^T, and *B. bifidum* YIT4039^T (= ATCC29521^T) grew in the presence of lactose and produced acetate and lactate at a ratio of approximately 3:2. *B. infantis* and *B. breve*, but not *B. bifidum*, produced acetate, formate, and 1,2-PD in the fucose-containing medium (Table S2). However, these three strains showed different phenotypes when cultured in medium containing 2′-FL (Fig. 6B and Table S2). The *B. infantis* strain grew in the medium, assimilating 2′-FL completely, and producing acetate, lactate, formate, and 1,2-PD. *B. bifidum* grew in the medium, produced acetate and lactate, and accumulated fucose in the supernatant. In contrast, *B. breve*, which lacks the FL transporter, was unable to grow in the medium containing 2′-FL. We subsequently co-cultured the *B. bifidum* and *B. breve* strains in 2′-FL medium to evaluate the substrate-cross-feeding, and confirmed our hypothesis that the combination of the strains could utilise the fucosylated HMO completely, and produce formate, 1,2-PD, acetate, and lactate.

Succinate-microbiota association in early life

We found positive association between Enterobacterales abundance and the succinate concentration with respect to age in some infants (Fig. S4 and S12), consistent with the report by Bittinger *et al.*[51]. On the other hand, less association was observed in the other infants, which implicate the involvement of the other bacterial taxa in gut succinate production. Previous study reported that gut microbes belonging to Bacteroidales (e.g. *Bacteroides thetaiotaomicron*) produce succinate[52]. Therefore, further studies are warranted to understand the involvement of other bacterial taxa, metabolic pathway, and substrate in early life gut succinate.

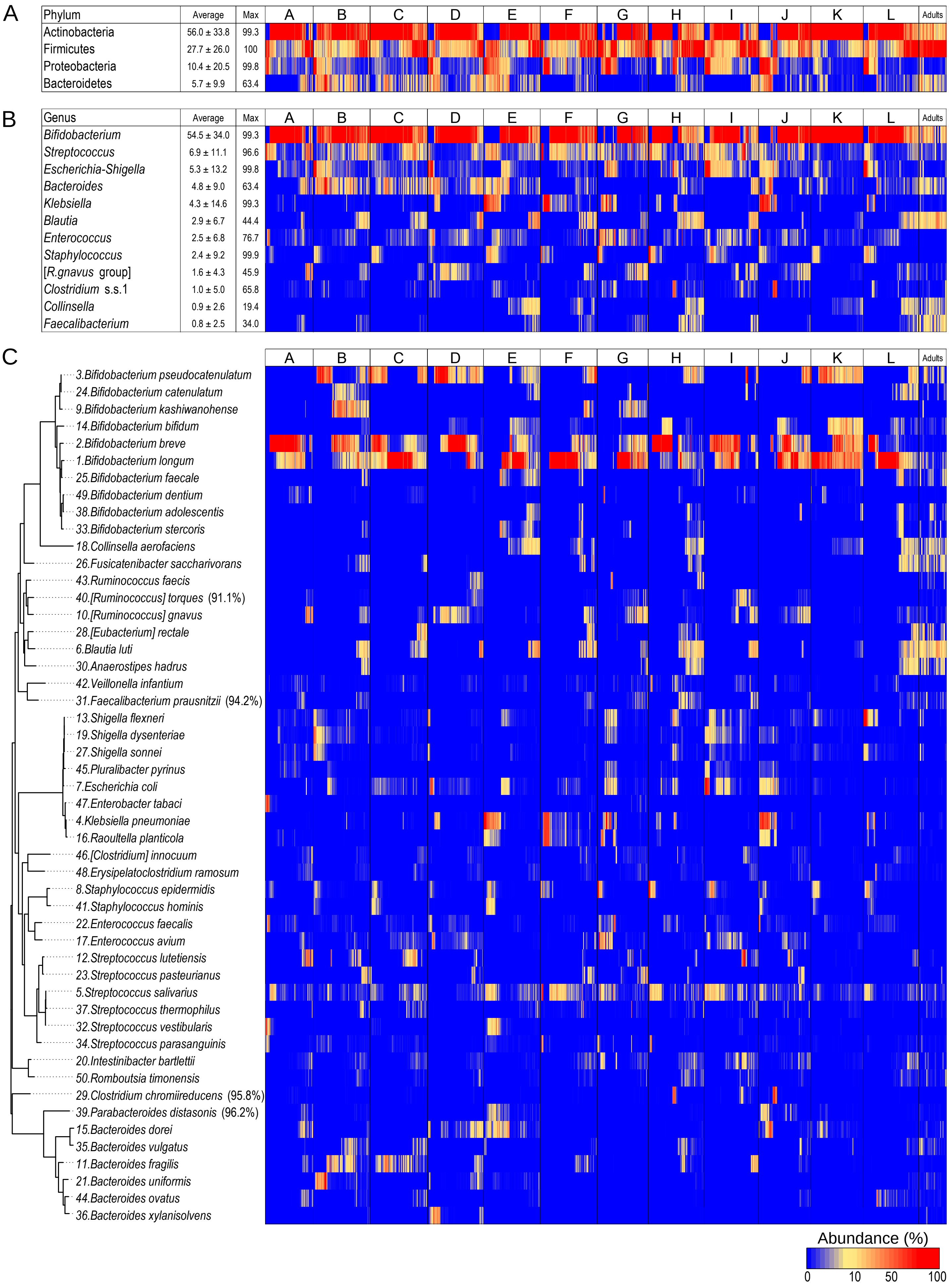

Fig. S1. Age-dependent heatmap at different taxonomic levels. Microbiota profiles of stool samples from 12 subjects are temporally ordered from left to right. **(A, B)** All phylum and top 12 genus heatmap and average abundance are shown. **(C)** The top 50 phylotypes are shown. Phylotype ID were allocated in average abundance order. Assigned species (or closest species with brackets showing % homology) are shown. Left tree represents the taxonomic relationship based on 16S rRNA sequences.

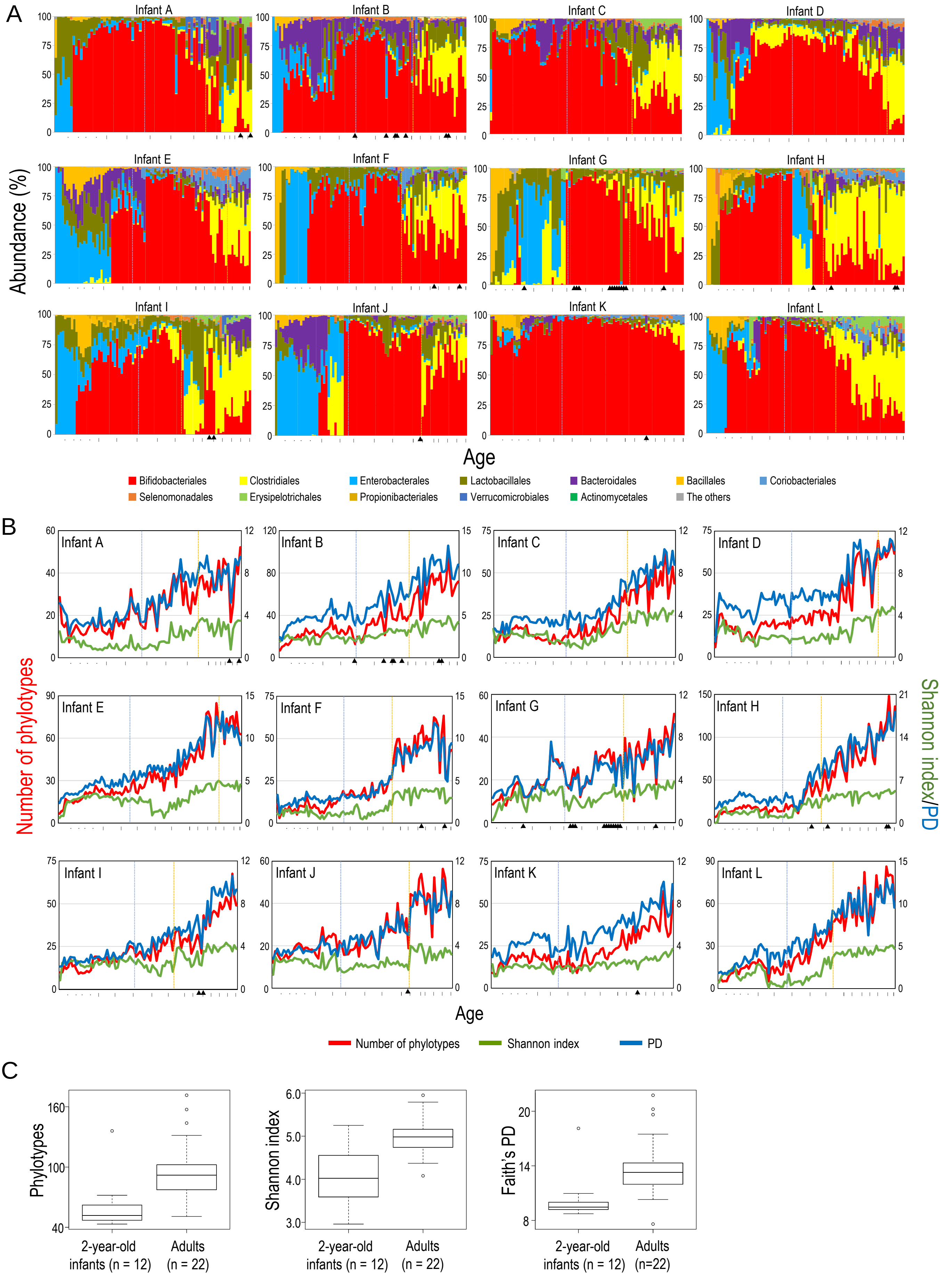

Fig. S2. Dynamics and inter-individual variation of gut microbiota development during the first two years of life. (A) Order-level dynamics of 12 infants are presented. Vertical bars along the x-axis indicate every two months. Dots along the x-axis indicate every week until one month. Blue and orange dotted vertical lines represent the initiation of solid food and cessation of breastfeeding, respectively. Black arrowheads along the x-axis indicate exposure to antibiotics. **(B)** Temporal shift of α-diversity during the first two years**.** Number of phylotypes, Shannon index, and Faith's phylogenetic diversity (PD) of 12 infants are presented. **(C)** Comparison of α-diversity between the two-year-old infants and adults. Number of phylotypes, Shannon index, and Faith's phylogenetic diversity (PD) of 12 infants and those of their parents (n = 22) are compared.

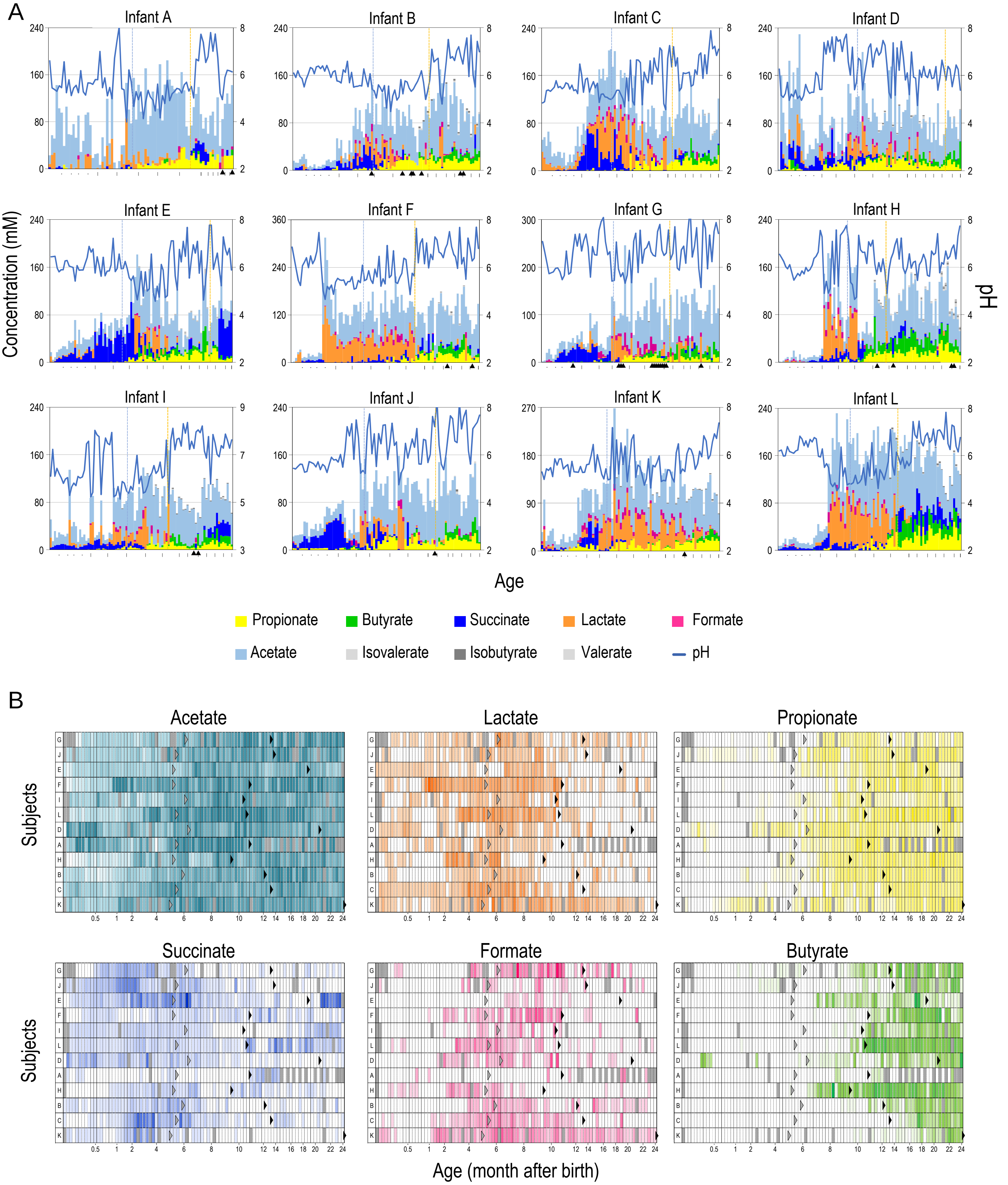

Fig. S3. Dynamics and inter-individual variation of gut SCFA profiles and pH. (A) Vertical bars along the x-axis indicate every two months. Dots along the x-axis indicate every week until one month. Blue and orange dotted vertical lines indicate the initiation of solid food and cessation of breastfeeding, respectively. Arrowheads along the x-axis indicate exposure to antibiotics. **(B)** Relationship between gut SCFAs and feeding. Gray and black arrowheads indicate the initiation of solid food and cessation of breastfeeding, respectively. Concentrations of gut SCFA were shown with respect to age in heatmap. Maximum concentration of acetate, propionate, butyrate, succinate, lactate, and formate were 183, 48, 61, 97, 140, and 31 mM, respectively. The subjects were ordered as in Fig. 1F.

SCFAs	Subjects	Bacterial order (abundance, average ± SD): Bifidobacteriales (55 ± 34%)	Clostridiales (12 ± 19%)	Enterobacterales (10 ± 21%)	Lactobacillales (10 ± 13%)	Bacteroidales (5.6 ± 9.9%)	Bacillales (2.5 ± 9.3%)
Butyrate	A	-0.22	0.36	-0.25	0.11	0.38	-0.11
	B	-0.20	0.79	-0.68	0.60	-0.36	-0.61
	C	-0.63	0.81	-0.39	0.25	0.12	-0.29
	D	-0.22	0.40	-0.19	0.41	0.16	-0.44
	E	0.57	0.25	-0.78	-0.30	-0.63	-0.55
	F	-0.33	0.90	-0.40	-0.05	0.77	-0.46
	G	0.21	0.62	-0.19	-0.25	0.42	-0.14
	H	-0.45	0.83	-0.20	-0.19	0.73	-0.49
	I	-0.41	0.84	-0.61	-0.04	0.56	-0.75
	J	-0.01	0.75	-0.35	0.13	-0.13	-0.25
	K	-0.30	0.84	-0.43	0.19	-0.19	-0.30
	L	-0.43	0.82	-0.47	-0.18	0.25	-0.19
Propionate	A	-0.37	0.82	-0.31	0.05	0.76	-0.28
	B	0.17	0.61	-0.69	0.56	-0.33	-0.73
	C	-0.64	0.70	-0.14	0.61	0.24	-0.42
	D	0.31	0.26	-0.45	0.26	-0.40	-0.47
	E	0.48	0.38	-0.85	-0.35	-0.51	-0.62
	F	-0.19	0.87	-0.37	-0.13	0.81	-0.54
	G	0.58	0.18	-0.50	-0.39	0.07	-0.36
	H	-0.38	0.84	-0.26	-0.22	0.70	-0.46
	I	-0.05	0.82	-0.62	-0.29	0.42	-0.77
	J	0.40	0.50	-0.50	0.17	-0.11	-0.54
	K	-0.06	0.71	-0.44	0.15	-0.02	-0.47
	L	-0.31	0.81	-0.62	-0.03	0.35	-0.40
Lactate	A	0.42	-0.29	-0.02	-0.24	-0.30	0.03
	B	0.53	-0.42	-0.04	-0.01	-0.28	0.06
	C	0.74	-0.72	-0.10	-0.45	-0.27	0.20
	D	0.31	-0.37	0.29	0.11	-0.33	0.11
	E	0.13	-0.55	0.33	0.03	-0.27	0.14
	F	0.70	-0.54	0.12	0.44	-0.47	0.00
	G	0.27	-0.32	-0.02	-0.16	-0.35	-0.17
	H	0.51	-0.54	0.14	-0.05	-0.37	-0.10
	I	0.52	-0.42	0.12	-0.10	-0.33	0.31
	J	0.40	-0.11	-0.11	0.02	-0.40	-0.21
	K	0.68	-0.15	-0.17	-0.24	-0.15	-0.29
	L	0.73	-0.45	-0.07	-0.02	0.19	-0.36
Formate	A	-0.09	0.07	-0.35	0.06	0.20	0.05
	B	0.67	-0.09	-0.45	0.19	-0.47	-0.21
	C	0.50	-0.39	-0.24	-0.21	-0.05	-0.26
	D	0.62	0.00	-0.27	-0.24	-0.58	-0.10
	E	0.59	-0.32	-0.22	-0.34	-0.52	-0.33
	F	0.78	-0.23	-0.15	0.04	-0.17	-0.36
	G	0.61	-0.39	-0.52	-0.22	-0.26	-0.35
	H	0.43	-0.51	0.12	0.01	-0.43	0.10
	I	0.57	-0.13	-0.04	-0.22	-0.27	0.05
	J	0.31	-0.02	-0.28	0.08	-0.39	-0.26
	K	0.69	-0.10	-0.36	-0.18	-0.11	-0.39
	L	0.62	-0.44	-0.16	-0.02	0.13	-0.43
Acetate	A	0.04	0.25	-0.15	-0.15	0.13	-0.13
	B	0.28	0.47	-0.69	0.47	-0.43	-0.70
	C	-0.18	0.30	-0.31	0.23	0.13	-0.40
	D	0.12	-0.18	0.19	0.38	-0.26	-0.14
	E	0.64	0.08	-0.66	-0.32	-0.58	-0.49
	F	0.34	0.38	-0.20	0.11	0.34	-0.47
	G	0.64	0.38	-0.49	-0.50	0.11	-0.38
	H	0.12	0.50	-0.10	-0.29	0.53	-0.55
	I	0.14	0.58	-0.45	-0.22	0.14	-0.62
	J	0.62	0.37	-0.52	0.25	-0.32	-0.70
	K	0.43	0.30	-0.40	-0.12	-0.10	-0.58
	L	0.34	0.29	-0.46	-0.11	0.45	-0.48
Succinate	A	-0.31	0.21	0.10	0.22	0.21	0.08
	B	0.03	-0.48	0.42	-0.31	0.24	0.33
	C	0.24	-0.42	0.22	-0.49	0.33	-0.10
	D	0.01	-0.35	0.33	-0.24	-0.05	0.67
	E	-0.17	0.01	0.30	0.11	0.56	0.16
	F	0.04	0.06	0.33	-0.35	0.04	-0.11
	G	-0.54	0.10	0.79	0.24	-0.01	0.08
	H	0.35	-0.11	0.31	-0.26	-0.14	-0.23
	I	-0.07	-0.25	0.16	-0.12	0.26	0.34
	J	-0.28	-0.57	0.53	-0.45	0.33	0.54
	K	-0.04	-0.54	0.33	-0.09	0.19	0.23
	L	-0.25	0.25	-0.20	-0.14	0.21	-0.16

Correlation scale: -1 / 0 / 1

Correlation

Fig. S4. Heatmap visualisation of within-subject correlation between gut SCFAs and bacterial abundance at order-level. Numbers represent *r* values for each infant (Spearman's correlation). FDR-corrected *p* values < 0.01 are underlined. Top 6 bacterial lineages are presented.

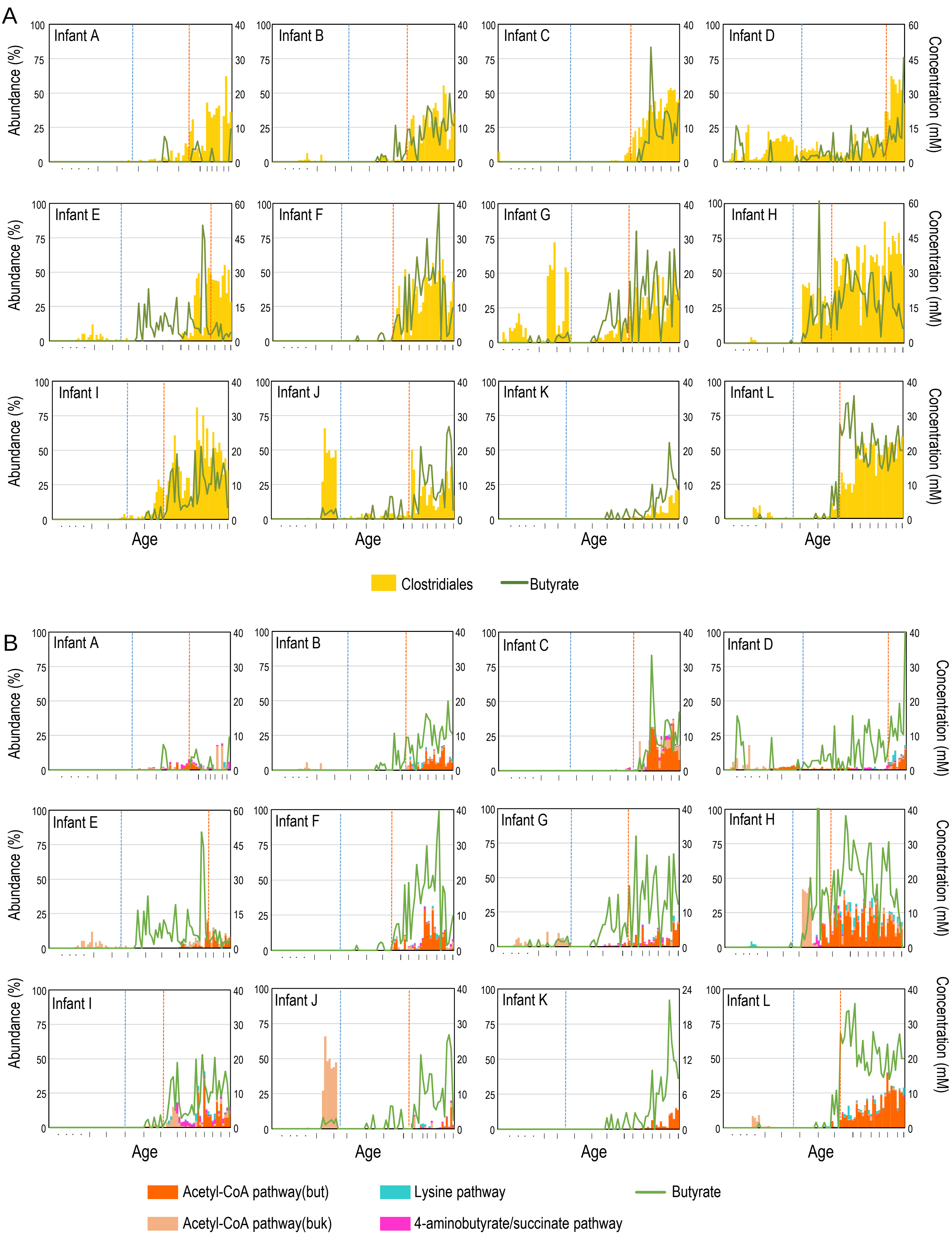

Fig. S5. Association between Clostridiales and gut butyrate. Vertical bars along the x-axis represent every two months. Dots along the x-axis indicate every week until one month. Blue and orange dotted vertical lines indicate the initiation of solid food and cessation of breastfeeding, respectively. **(A)** Clostridiales abundance and butyrate concentrations with respect to age. **(B)** Abundance of potent butyrate producing Clostridiales and faecal butyrate concentration with respect to age. The Clostridiales phylotypes having any pathway(s) for butyrate production are represented as stacked bar graphs.

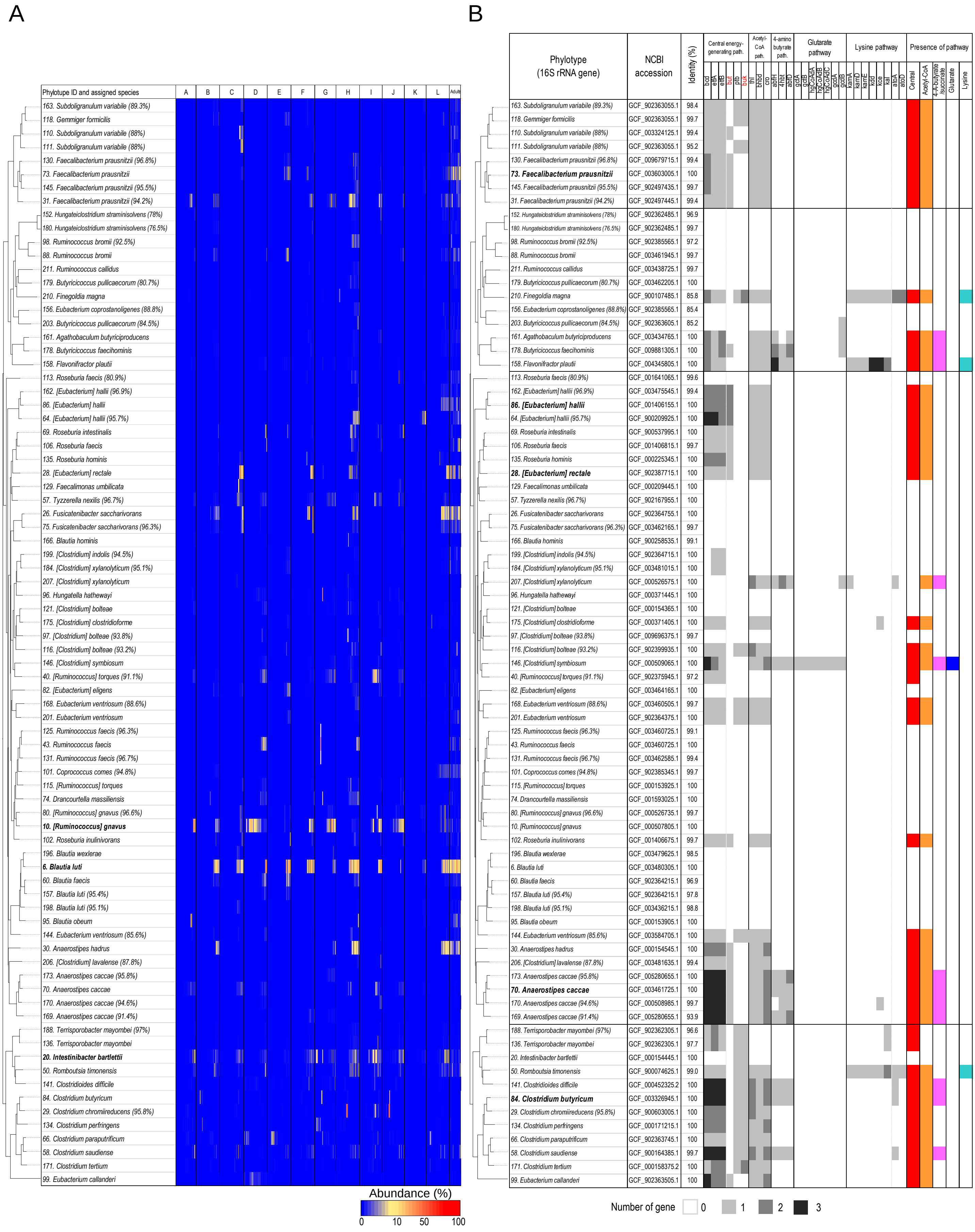

Fig. S6. Diverse and personalised Clostridiales phylotypes contribute to gut butyrate production. (A) Age-dependent heatmap of gut Clostridiales phylotypes. Phylotype IDs are allocated in average abundance order for all phylotypes; only the top 80 Clostridiales phylotypes are illustrated. Phylotype ID and assigned species (or the closest species with % homology in brackets) are shown. Left tree represents the taxonomic relationship based on 16S rRNA sequences. The samples from 12 subjects were temporally ordered from left to right. **(B)** Potential for butyrate production of each phylotype. Presence of genes (gray scales) and the pathway (colored) associated with butyrate production found in top 80 phylotypes were shown. See ref. (35) for abbreviations.

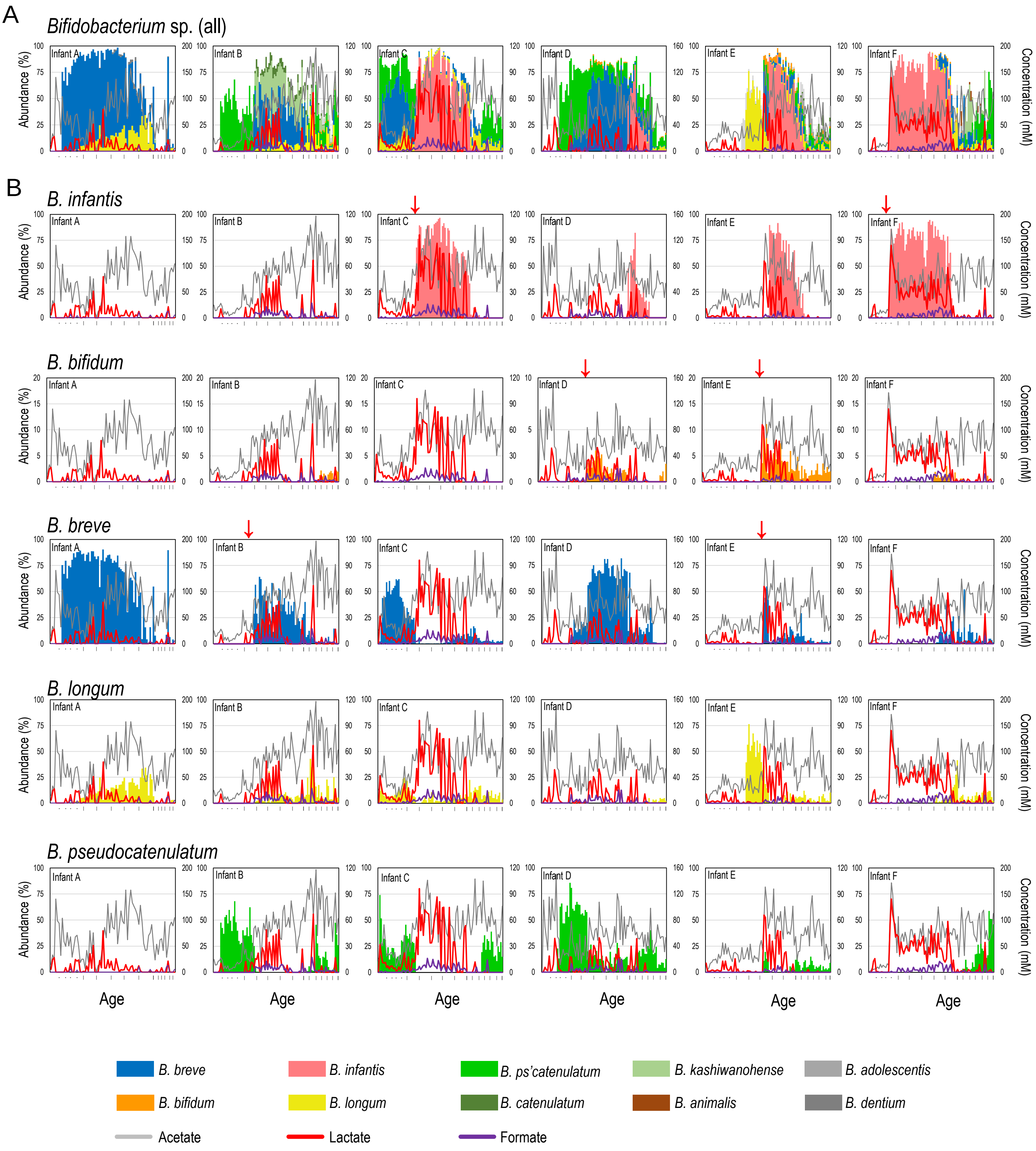

Fig. S7 (Subjects A to F). *Bifidobacterium* species abundance and SCFA concentrations with respect to age. **(A)** All bifidobacterial species. **(B)** Each bifidobacterial species. Red arrow above the graph represent the increase in SCFA concentration coupled with increased bifidobacterial species abundance. Vertical bars along the x-axis represent every two months. Dots along the x-axis indicate every week until one month.

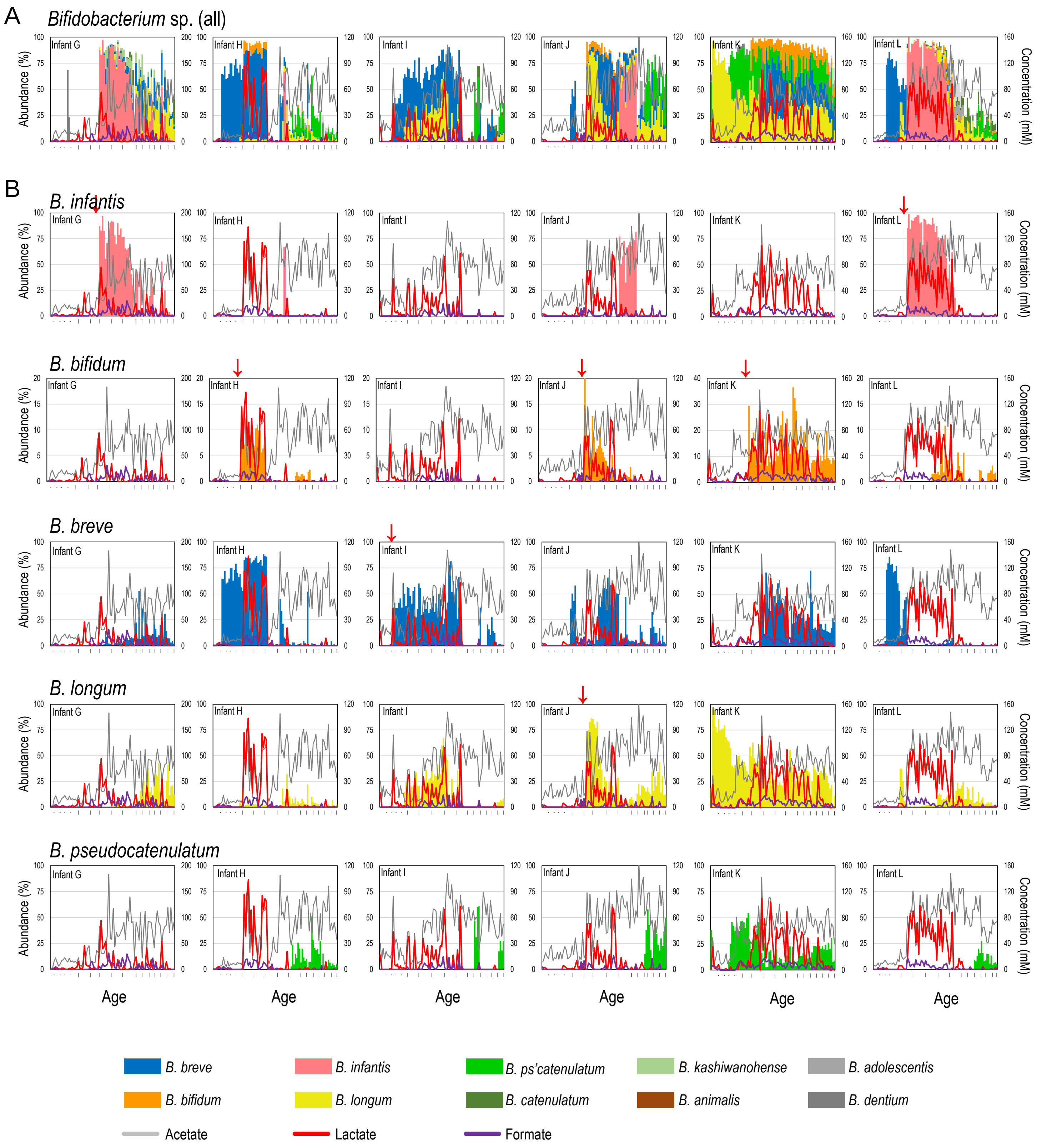

Fig. S7 (Subjects G to L). *Bifidobacterium* species abundance and SCFA concentrations with respect to age. **(A)** All bifidobacterial species. **(B)** Each bifidobacterial species. Red arrow above the graph represent the increase in SCFA concentration coupled with increased bifidobacterial species abundance. Vertical bars along the x-axis represent every two months. Dots along the x-axis indicate every week until one month.

SCFAs	Subjects	Bifidobacteriales	*B. infantis*	*B. bifidum*	*B. breve*	*B. longum*	*B. pseudocatenulatum*	*B. catenulatum*	*B. kashiwanohense*	*B. dentium*	*B. adolescentis*	*B. faecale*	*B. animalis*
Lactate	A	0.42	NA	0.16	0.39	0.14	NA	NA	-0.12	-0.07	NA	NA	NA
	B	0.53	NA	-0.18	0.41	0.18	-0.35	0.44	0.45	-0.03	NA	-0.12	NA
	C	0.74	0.52	NA	-0.28	-0.32	-0.29	NA	NA	-0.59	NA	NA	NA
	D	0.31	-0.13	0.34	0.17	-0.42	-0.22	NA	NA	-0.15	-0.03	-0.10	NA
	E	0.13	0.22	0.11	-0.05	-0.34	-0.23	-0.44	NA	-0.25	-0.47	-0.64	-0.14
	F	0.70	0.77	0.05	-0.30	-0.38	-0.50	-0.22	-0.42	0.00	-0.22	-0.22	-0.41
	G	0.27	0.22	0.10	0.08	-0.01	-0.23	-0.36	0.06	-0.16	NA	NA	NA
	H	0.51	-0.22	0.58	0.48	0.04	-0.36	NA	NA	-0.09	-0.27	-0.26	-0.12
	I	0.52	NA	-0.09	0.44	0.49	-0.19	-0.26	NA	-0.29	NA	NA	-0.37
	J	0.40	-0.08	0.53	0.35	0.32	-0.41	NA	NA	-0.15	0.00	NA	0.19
	K	0.68	NA	0.60	0.53	-0.34	-0.05	-0.04	NA	-0.10	-0.36	-0.40	0.01
	L	0.73	0.86	0.12	0.17	0.05	-0.43	-0.30	-0.11	NA	-0.26	-0.26	NA
Formate	A	-0.09	NA	-0.04	0.02	-0.15	NA	NA	-0.04	0.08	NA	NA	NA
	B	0.67	NA	0.03	0.64	0.49	-0.53	0.70	0.70	0.02	NA	0.03	NA
	C	0.50	0.73	NA	-0.50	-0.44	-0.50	NA	NA	-0.29	NA	NA	NA
	D	0.62	-0.06	0.32	0.47	-0.25	0.25	NA	NA	0.01	0.17	0.08	NA
	E	0.59	0.49	0.53	0.45	0.12	0.23	-0.17	NA	-0.11	-0.17	-0.17	-0.06
	F	0.78	0.68	0.34	0.03	-0.06	-0.34	-0.05	-0.29	-0.08	-0.09	-0.09	-0.28
	G	0.61	0.54	0.26	0.26	0.12	0.12	0.11	0.38	-0.21	NA	NA	NA
	H	0.43	-0.26	0.47	0.44	-0.14	-0.43	NA	NA	-0.20	-0.37	-0.24	0.07
	I	0.57	NA	-0.06	0.32	0.53	-0.13	-0.11	NA	-0.19	NA	NA	-0.25
	J	0.31	0.01	0.31	0.25	0.24	-0.12	NA	NA	-0.09	-0.17	NA	0.25
	K	0.69	NA	0.39	0.44	-0.33	0.10	-0.12	NA	0.07	-0.29	-0.37	0.09
	L	0.62	0.75	-0.09	-0.08	-0.16	-0.27	-0.32	-0.09	NA	-0.36	-0.34	NA

Correlation

-1 0 1

Fig. S8. Heatmap visualisation of within-individual Spearman's correlations between *Bifidobacterium* species and lactate and formate concentrations. FDR-corrected *p* values < 0.01 are underlined.

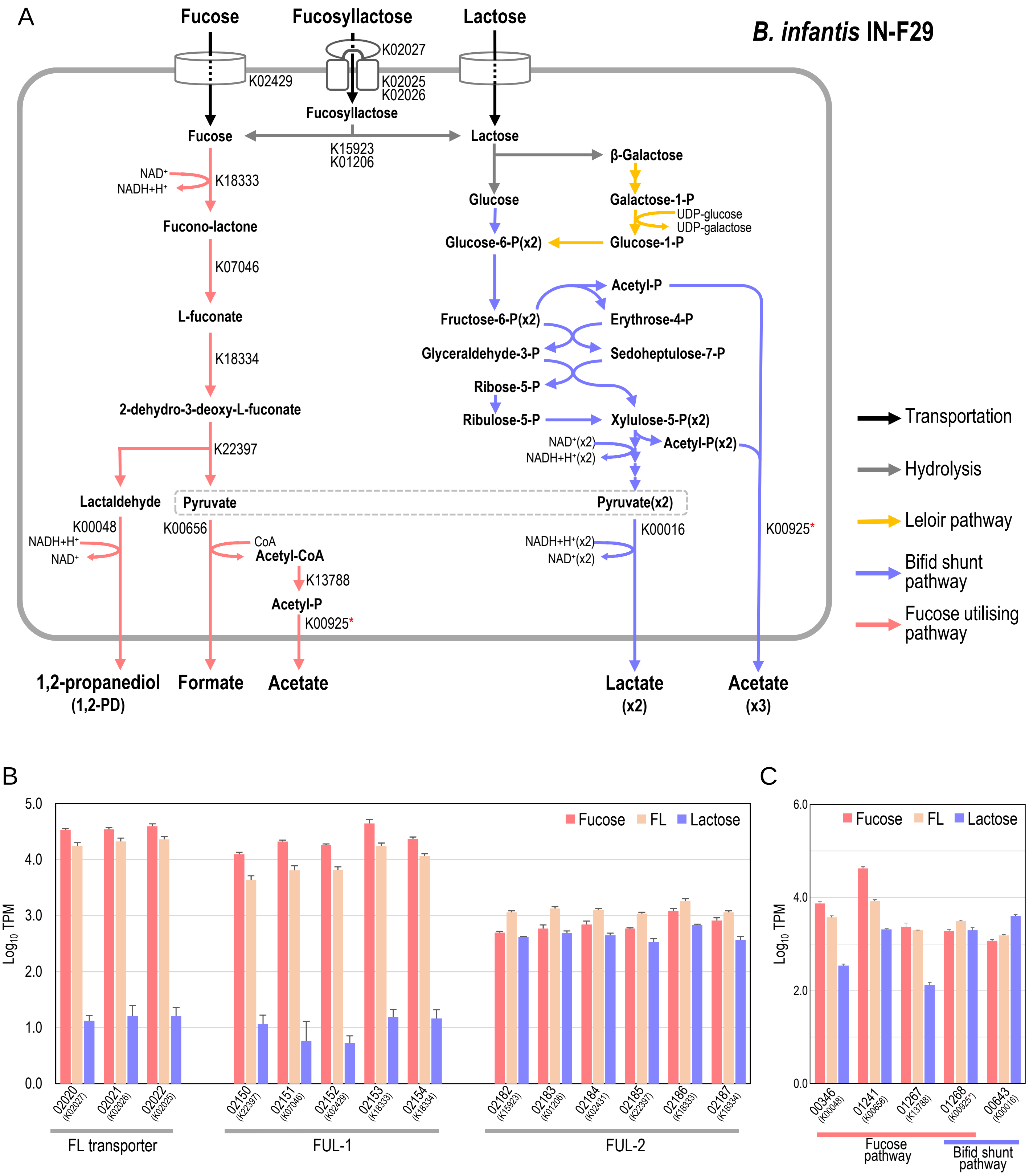

Fig. S9. Bifidobacterial metabolic pathways to produce formate and its substrate. (A) Schematic representation of the pathway for the utilisation of fucosyllactose, lactose, and fucose in *B. infantis*. **(B)** RNA expression profile during utilisation of fucose, FL, and lactose in *B. infantis* strain IN-F29. Expression of the genes located in ABC transporter for FL, FUL-1, and FUL-2 are shown. Number represent locus tags (INF29_xxxxx are abbreviated). **(C)** Expression of genes involved in SCFAs and 1,2-PD production during growth in fucose, FL, and lactose. Only the genes in the final step were focused. *Proteins annotated as K00925 are included in both fucose metabolising pathway and Bifid shunt pathway.

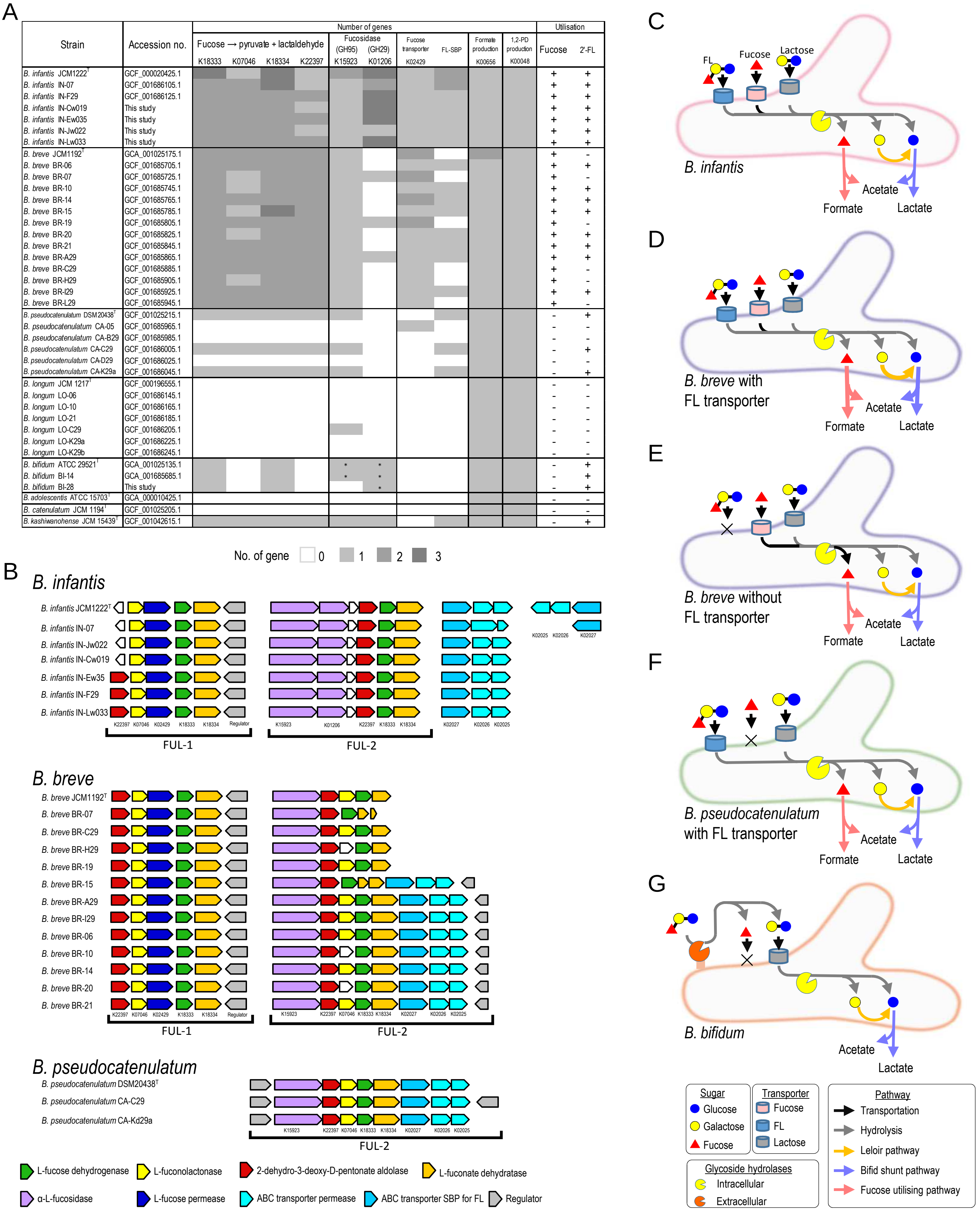

Fig. S10. Genes and loci involved in fucose and fucosylated HMO utilisation and their variation among bifidobacterial species. (A) Distribution of fucose-metabolizing genes in representative human gut bifidobacteria. The number of categorized genes is presented. Functional assignment was based on BlastKOALA, except for ABC transporter SBP for FL (FL-SBP), which was annotated using BLASTn with a reference sequence (LC068768). *, fucosidase of *B. bifidum* was predicted to be extracellular, whereas that of the other bifidobacteria were predicted as intracellular. **(B)** Gene conservation and organisation. Strains without the loci are not shown. **(C-G)** Utilisation of fucosyllactose by infant bifidobacteria. **(C, D)** *B. infantis* and *B. breve* with FL-SBP utilise fucose and FL. **(E)** *B. breve* without FL-SBP utilise fucose but not FL. **(F)** *B. pseudocatenulatum* with FL-SBP utilise FL but not extracellular fucose. **(G)** *B. bifidum* that possess extracellular fucosidase digest FL into fucose and lactose extracellularly. The species uptake and utilise the resultant lactose but not fucose.

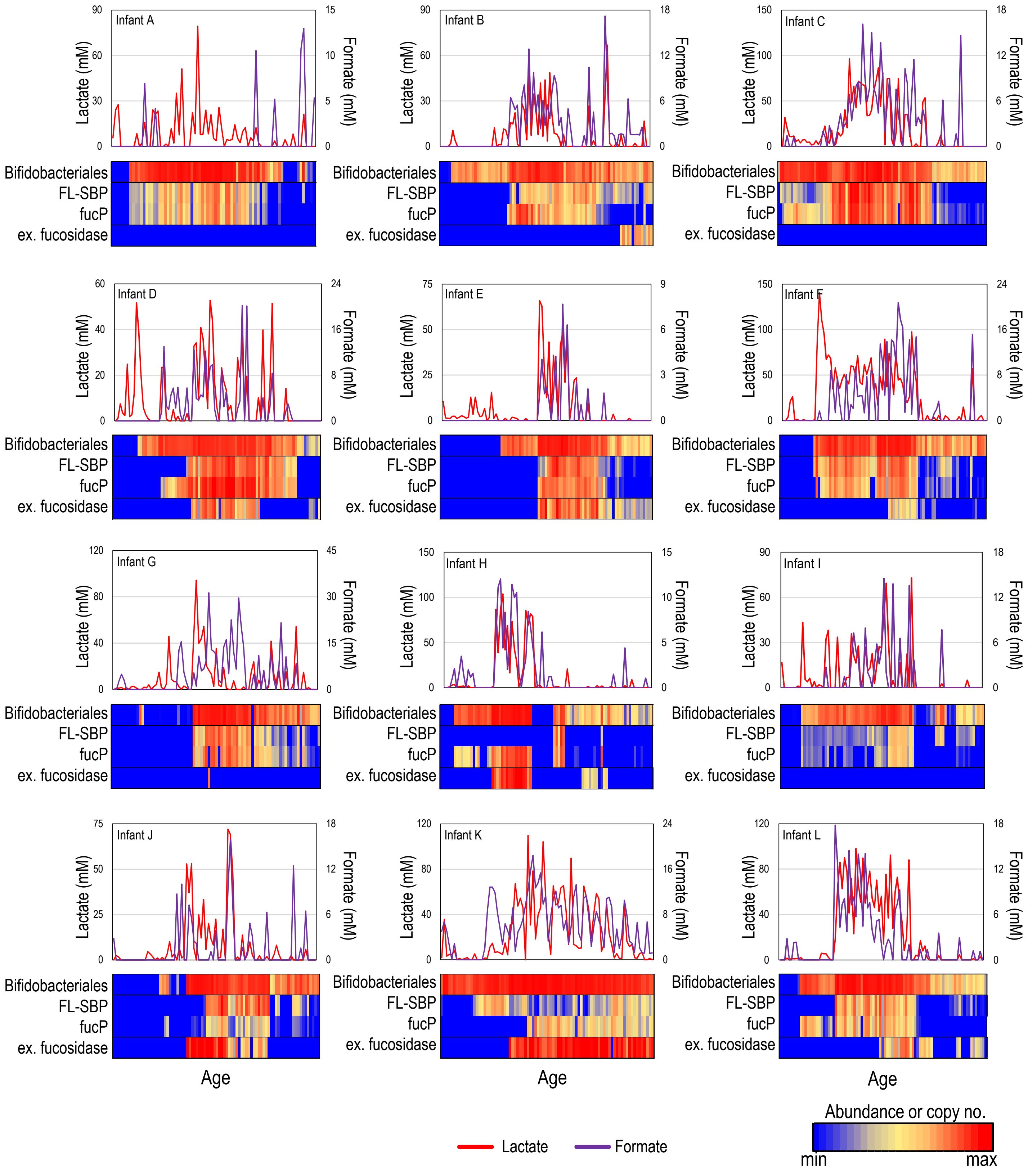

Fig. S11. Relationship between key marker genes for fucosyl HMO utilisation and concentration of lactate and formate. The concentration of lactate and formate are shown in the line graph, whereas the abundance of Bifidobacteriales and the presence of the key marker genes are visualised in heatmap. Color scale of Bifidobacteriales ranged from 0 to 100%, FL-SBP and fucose symporter (fucP) ranged from 10^9 to 10^{11} copies per g of feces (log scale), and extracellular fucosidase (denoted as ex. fucosidase) ranged from 10^8 to 10^{10} copies per g of feces (log scale).

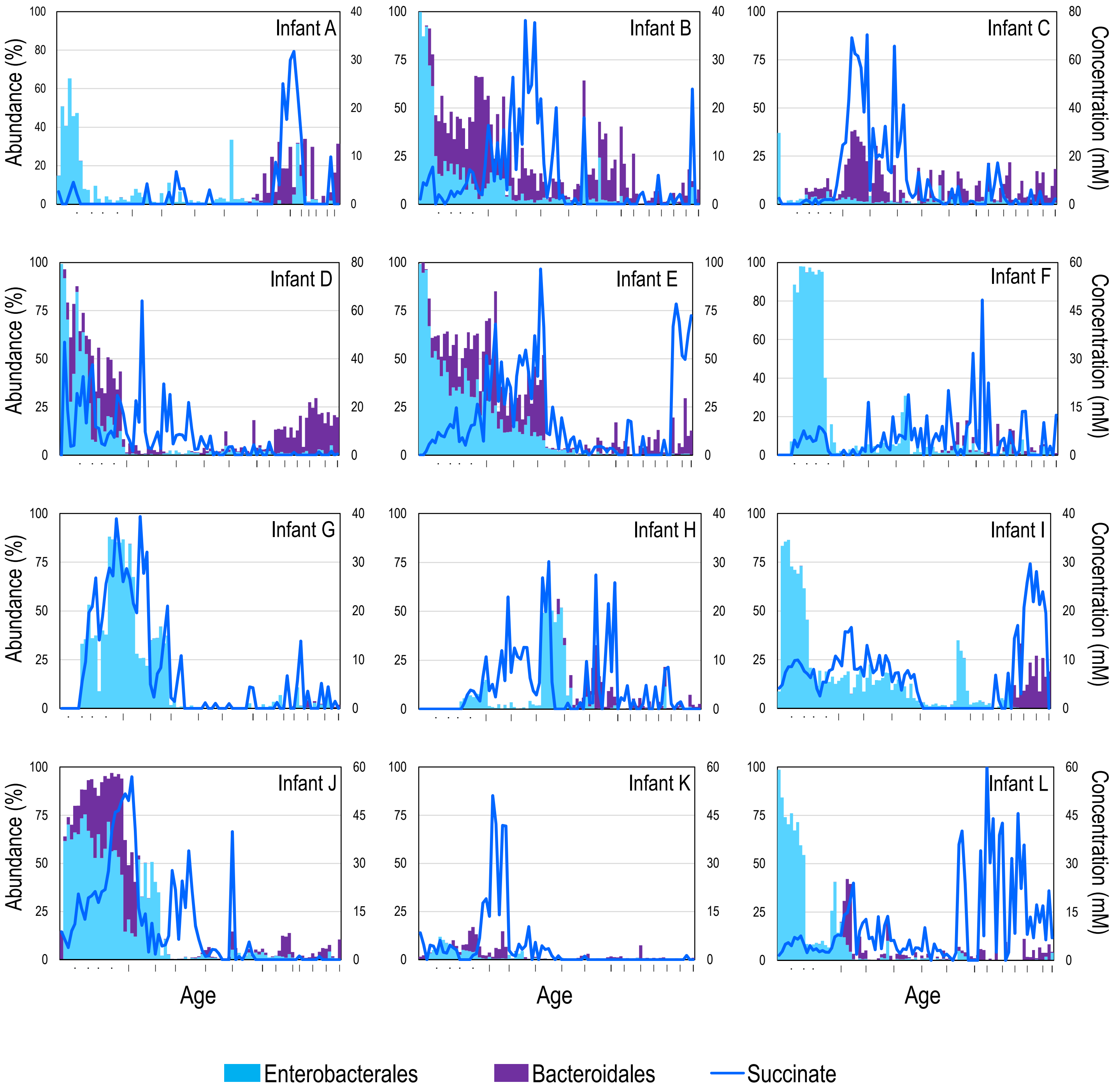

Fig. S12. Relationship between gut succinate concentration and the abundance of Enterobacterales and Bacteroidetes with respect to age. See Supplementary text for Succinate-Bacteroidales relationship. Vertical bars along the x-axis represent every two months. Dots along the x-axis indicate every week until one month.

	Subjects	SCFA						Microbiota					
		Acetate	Lactate	Succinate	Propionate	Butyrate	Formate	Bifidobacteriales	Clostridiales	Enterobacterales	Lactobacillales	Bacteroidales	Bacillales
pH	A	-0.49	-0.49	0.24	0.12	0.15	-0.08	-0.40	0.16	0.17	0.31	0.21	0.10
	B	-0.16	-0.65	-0.18	0.07	0.50	-0.45	-0.54	0.51	-0.21	0.25	-0.06	-0.06
	C	-0.07	-0.82	-0.34	0.66	0.43	-0.42	-0.61	0.63	0.01	0.41	0.32	-0.29
	D	-0.42	-0.41	-0.24	0.59	0.03	0.07	0.34	0.36	-0.59	-0.13	-0.37	-0.20
	E	-0.45	-0.49	-0.16	0.14	-0.15	-0.46	-0.36	0.44	-0.03	0.24	0.12	0.10
	F	-0.11	-0.85	0.11	0.68	0.66	-0.39	-0.49	0.67	-0.18	-0.40	0.55	-0.16
	G	-0.01	-0.53	-0.09	0.17	0.47	-0.14	-0.11	0.19	0.00	0.05	0.40	-0.06
	H	-0.30	-0.31	0.19	0.24	0.11	-0.23	-0.32	0.29	0.03	-0.03	0.11	-0.11
	I	-0.11	-0.68	-0.08	0.35	0.55	-0.30	-0.33	0.43	-0.29	0.17	0.28	-0.37
	J	0.11	-0.33	-0.34	0.57	0.49	-0.03	0.29	0.72	-0.42	-0.08	-0.40	-0.39
	K	-0.16	-0.59	0.04	0.69	0.49	-0.24	-0.22	0.49	-0.19	0.10	-0.21	-0.21
	L	-0.39	-0.82	0.27	0.44	0.48	-0.68	-0.62	0.55	-0.17	0.09	-0.23	0.13

Correlation: -1 / 0 / 1

Fig. S13. Heatmap visualisation of within-subject correlation between gut pH-SCFA and pH-microbiota. Numbers represent *r* values (Spearman's correlation). FDR-corrected *p* values < 0.01 are underlined.

Table S1. Relevant characteristics of infants, whose gut microbiota were investigated during the first two years of life*

Subject ID	Delivery	Birth weight (g)	Sex	Gestation (week)	Initiation of solid food (month)	Cessation of breast-feeding (month)	Antibiotic exposure (week)	First probiotic exposure (week)		Number of samples	Age of parents		Furry pet exposure (month)	Number of sibling
								Bifidobacteria	Other genera		Father	Mother		
A	Vaginal	2,820	M	40	5.4	10.9	84, 98	Not recorded	Not recorded	77	42	41	Not exposed	0
B	Vaginal	3,030	F	40	5.8	12.7	25, 40, 44, 45 49, 84, 86	Not recorded	54	91	43	42	0-24	1
C	Vaginal	2,620	M	39	5.5	13.3	Not exposed	56	56	92	29	n.t.	Not exposed	0
D	Vaginal	2,750	F	38	6.3	20.4	Not exposed	58	40	90	36	33	Not exposed	1
E	Vaginal	2,544	F	40	5.2	18.8	Not exposed	46	64	91	33	31	Not exposed	0
F	Vaginal	2,706	M	38	5.2	11.0	74, 98	37	30	91	31	29	Not exposed	0
G	Vaginal	2,525	M	38	6.1	13.5	5, 29-31, 46-54, 88	17	19	82	33	n.t.	Not exposed	0
H	Vaginal	2,935	M	38	5.2	9.6	37, 45, 96-98	54	43	90	29	30	0-9	1
I	Vaginal	2,926	M	40	6.0	10.4	66, 70	44	17	87	31	30	Not exposed	0
J	Vaginal	3,178	F	38	5.3	13.6	58	62	62	84	31	31	Not exposed	1
K	Vaginal	3,092	F	38	4.9	24.0	70	Not recorded	Not recorded	84	34	34	Not exposed	0
L	Vaginal	2,960	M	39	5.5	10.8	Not exposed	47	45	89	35	33	Not exposed	1

* Infant A and B are sibling. Infant E and L are sibling.

Table S2. Utilisation of HMO-derived carbohydrate and the accumulation of SCFA, fucose and 1,2-PD

Species	Substrate Supplied (%)	Growth	Remainig suger in culture supernatant(%)			Metabolite (mM)			
		OD_{600}	Lactose	Fucose	2'-FL	Acetate	Lactate	Formate*	1,2-PD
B. infantis	Lactose (0.5%)	1.0	0.0	0.0	0.0	41.5	22.2	2.7	0.0
	Fucose (1%)	0.3	0.0	0.5	0.0	17.0	1.7	17.8	27.0
	2'-FL (0.5%)	0.4	0.0	0.0	0.0	38.9	15.4	11.6	7.8
B. breve	Lactose (0.5%)	1.1	0.0	0.0	0.0	40.5	23.3	1.2	0.0
	Fucose (1%)	0.1	0.0	0.3	0.0	21.9	1.6	22.5	41.0
	2'-FL (0.5%)	0.0	0.0	0.0	0.6	2.2	0.1	0.2	0.0
B. bifidum	Lactose (0.5%)	0.5	0.0	0.0	0.0	40.6	22.3	2.9	0.0
	Fucose (1%)	0.0	0.0	1.1	0.0	0.2	0.2	0.0	0.0
	2'-FL (0.5%)	1.1	0	0.2	0	37	19	2	0
B. breve and *B. bifidum*	Lactose (0.5%)	0.3	0.0	0.0	0.0	42.3	22.4	3.2	0.0
	Fucose (1%)	0.0	0.0	0.9	0.0	2.7	0.4	2.6	6.0
	2'-FL (0.5%)	0.5	0.0	0.1	0.0	41.9	7.3	15.1	5.9

* Formate production by these bifidobacteria is visualised in Fig. 6B.

Table S3. Specific PCR primers used in this study

Target	Primer	Sequence	Size (bp)	Tm	References
B. longum ss. *infantis* (specific co-transporter)	Binf1-F<br>Binf1-R	AGCAGCAGAAGTCCAGTGAAG<br>AGTAGTGGATGGTCGGCATAC	156	64°C	This study
B. longum ss. *longum* (16S)	s-Blon182-F<br>s-Blon1008-R	TTCCAGTTGATCGCATGGTC<br>GGGAAGCCGTATCTCTACGA	831	55°C	Matsuki et. al 2004
ABC transporter SBP for FL	Bif_flsbp271-F<br>Bif_flsbp754-R	GAYCATTTCACSAARTTCCAGAAYGTYG<br>YGGRAKATAMTCCCAATCSGTRTTSAC	510	58°C	This study
Fucose permease	Bif_fucP895-F<br>Bif_fucP1264-R	CGMTTCGYCACCACCC<br>GCCTCCTTCAGACCGAT	386	55°C	This study
Extracelullar fucosidase (GH29)	sBbif_GH29_3579-F<br>sBbif_GH29_3784-R	CGACGGCAACAATAACGG<br>GGCAGCAACTCGACATTCAT	227	55°C	This study

Supplementary Data 1. Information regarding sample backgrounds (subjects, age, feeding, and exposure to antibiotics and probiotics), 16S rRNA gene amplicon accession number, SCFA concentrations, and pH.

Sample ID	Sub-jects	Age		Feeding	Exposure		SCFA									pH	16S amplicon
		day	month	(6 types)	Antibiotics	Probiotics	Acetate	Lactate	Succinate	Propionate	Butyrate	Formate	Isovalerate	Isobutyrate	Valerate		accession no.
Ad002	A	2	0.07	Mixed-milk	no	no	14.5	5.7	2.6	0.0	0.0	0.0	0.0	0.0	0.0	6.6	SAMD00242836
Ad003	A	3	0.10	Mixed-milk	no	no	33.1	24.0	0.0	0.0	0.0	0.0	0.0	0.0	0.0	5.4	SAMD00242835
Ad004	A	4	0.13	Mixed-milk	no	no	24.2	27.6	0.0	0.0	0.0	0.0	0.0	0.0	0.0	5.4	SAMD00242837
Ad005	A	5	0.16	Mixed-milk	no	no	140.4	0.0	2.2	0.0	0.0	0.0	0.0	0.0	0.0	5.3	SAMD00242838
Ad006	A	6	0.20	Mixed-milk	no	no	98.6	0.0	4.5	0.0	0.0	0.0	0.0	0.0	0.0	5.5	SAMD00242839
Ad007	A	7	0.23	Mixed-milk	no	no	93.7	0.0	2.1	0.0	0.0	0.0	0.0	0.0	0.0	5.5	SAMD00242840
Ad009	A	9	0.30	Mixed-milk	no	no	31.9	0.0	0.0	7.1	0.0	0.0	0.0	0.0	0.0	6.6	SAMD00242841
Ad011	A	11	0.36	Mixed-milk	no	no	103.7	0.0	0.0	2.1	0.0	0.0	0.0	0.0	0.0	5.2	SAMD00242842
Ad013	A	13	0.43	Mixed-milk	no	no	58.9	0.0	0.0	0.0	0.0	0.0	0.0	0.0	0.0	5.8	SAMD00242843
Ad015	A	15	0.49	Breast-milk	no	no	49.3	8.5	0.0	0.0	0.0	0.0	0.0	0.0	0.0	5.1	SAMD00242844
Ad017	A	17	0.56	Breast-milk	no	no	76.0	0.0	0.0	0.0	0.0	0.0	0.0	0.0	0.0	5.4	SAMD00242845
Ad019	A	19	0.62	Breast-milk	no	no	50.3	2.4	0.0	0.0	0.0	0.0	0.0	0.0	0.0	5.6	SAMD00242846
Ad021	A	21	0.69	Breast-milk	no	no	67.7	16.0	0.0	0.0	0.0	6.9	0.0	0.0	0.0	5.2	SAMD00242847
Ad023	A	23	0.76	Breast-milk	no	no	28.9	0.0	0.0	0.0	0.0	0.0	0.0	0.0	0.0	6.2	SAMD00242848
Ad025	A	25	0.82	Breast-milk	no	no	47.6	0.0	0.0	0.0	0.0	0.0	0.0	0.0	0.0	5.3	SAMD00242849
Ad027	A	27	0.89	Breast-milk	no	no	67.9	24.2	0.0	0.0	0.0	0.0	0.0	0.0	0.0	5.1	SAMD00242850
Ad029	A	29	0.95	Breast-milk	no	no	70.7	21.2	0.0	0.0	0.0	4.1	0.0	0.0	0.0	5.7	SAMD00242851
Aw005	A	37	1.22	Breast-milk	no	no	51.2	23.4	0.0	0.0	0.0	0.0	0.0	0.0	0.0	5.7	SAMD00242852
Aw006	A	44	1.45	Breast-milk	no	no	18.4	0.0	0.0	0.0	0.0	0.0	0.0	0.0	0.0	5.6	SAMD00242853
Aw007	A	51	1.68	Breast-milk	no	no	25.2	0.0	0.0	0.0	0.0	0.0	0.0	0.0	0.0	5.8	SAMD00242854
Aw008	A	58	1.91	Breast-milk	no	no	24.7	1.6	0.0	0.0	0.0	0.0	0.0	0.0	0.0	5.8	SAMD00242855
Aw009	A	65	2.14	Breast-milk	no	no	6.9	0.0	0.0	0.0	0.0	0.0	0.0	0.0	0.0	7.0	SAMD00242856
Aw010	A	72	2.37	Breast-milk	no	no	41.6	11.8	0.0	0.0	0.0	0.0	0.0	0.0	0.0	5.5	SAMD00242857
Aw011	A	79	2.60	Breast-milk	no	no	19.0	2.2	0.0	0.0	0.0	0.0	0.0	0.0	0.0	5.9	SAMD00242858
Aw012	A	86	2.83	Breast-milk	no	no	79.8	35.1	4.2	0.0	0.0	0.0	0.0	0.0	0.0	5.1	SAMD00242859
Aw013	A	93	3.06	Breast-milk	no	no	35.7	7.6	0.0	0.0	0.0	0.0	0.0	0.0	0.0	5.5	SAMD00242860
Aw014	A	100	3.29	Breast-milk	no	no	75.5	51.2	0.0	0.0	0.0	0.0	0.0	0.0	0.0	5.0	SAMD00242861
Aw016	A	109	3.58	Breast-milk	no	no	10.7	0.0	0.0	0.0	0.0	0.0	0.0	0.0	0.0	6.7	SAMD00242862
Aw017	A	121	3.98	Breast-milk	no	no	0.7	0.0	0.0	0.0	0.0	0.0	0.0	0.0	0.0	7.4	SAMD00242863
Aw018	A	128	4.21	Breast-milk	no	no	13.1	0.0	0.0	0.0	0.0	0.0	0.0	0.0	0.0	8.0	SAMD00242864
Aw019	A	135	4.44	Breast-milk	no	no	14.9	22.3	2.5	0.0	0.0	0.0	0.0	0.0	0.0	5.4	SAMD00242865
Aw020	A	143	4.70	Breast-milk	no	no	31.7	8.4	0.0	0.0	0.0	0.0	0.0	0.0	0.0	5.4	SAMD00242866
Aw021	A	151	4.96	Breast-milk	no	no	60.2	79.3	6.8	0.0	0.0	0.0	0.0	0.0	0.0	4.0	SAMD00242867
Aw022	A	157	5.16	Breast-milk	no	no	85.8	7.9	3.1	4.0	0.0	0.0	0.0	0.0	0.0	6.2	SAMD00242868
Aw023	A	163	5.36	Breast-milk	no	no	66.0	8.0	3.3	0.0	0.0	0.0	0.0	0.0	0.0	5.7	SAMD00242869
Aw024	A	170	5.59	milk & solid	no	no	131.2	23.9	0.0	0.0	0.0	0.0	0.0	0.0	0.0	4.7	SAMD00242870
Aw025	A	178	5.85	milk & solid	no	no	75.4	8.3	0.0	0.0	0.0	0.0	0.0	0.0	0.0	5.3	SAMD00242871
Aw026	A	185	6.08	milk & solid	no	no	113.3	20.9	0.0	8.8	0.0	0.0	0.0	0.0	0.0	4.9	SAMD00242872
Aw027	A	190	6.25	milk & solid	no	no	85.9	5.2	0.0	6.4	0.0	0.0	0.0	0.0	0.0	5.3	SAMD00242873
Aw028	A	198	6.51	milk & solid	no	no	82.4	3.8	0.0	3.0	0.0	0.0	0.0	0.0	0.0	4.1	SAMD00242874
Aw029	A	205	6.74	milk & solid	no	no	137.1	25.7	0.0	8.3	0.0	0.0	0.0	0.0	0.0	4.8	SAMD00242875
Aw030	A	212	6.97	milk & solid	no	no	54.5	12.2	3.0	6.1	0.0	0.0	0.0	0.0	0.0	5.3	SAMD00242876
Aw031	A	219	7.20	milk & solid	no	no	84.8	0.0	0.0	5.0	0.0	0.0	0.0	0.0	0.0	5.1	SAMD00242877
Aw032	A	226	7.43	milk & solid	no	no	117.6	4.6	0.0	9.3	0.0	0.0	0.0	0.0	0.0	4.9	SAMD00242878
Aw033	A	233	7.66	milk & solid	no	no	97.9	3.9	0.0	5.7	0.0	0.0	0.0	0.0	0.0	4.5	SAMD00242879
Aw034	A	240	7.89	milk & solid	no	no	93.1	4.7	0.0	4.8	0.0	0.0	0.0	0.0	0.0	5.6	SAMD00242880
Aw035	A	249	8.19	milk & solid	no	no	157.5	14.4	0.0	6.3	0.0	0.0	0.0	0.0	0.0	4.2	SAMD00242881
Aw036	A	255	8.38	milk & solid	no	no	62.3	5.7	0.0	14.1	0.0	0.0	0.0	0.0	0.0	5.1	SAMD00242882
Aw037	A	261	8.58	milk & solid	no	no	124.5	2.4	0.0	9.2	7.4	0.0	0.0	0.0	0.0	4.8	SAMD00242883
Aw038	A	269	8.84	milk & solid	no	no	157.5	9.9	0.0	11.6	5.8	0.0	0.0	0.0	0.0	5.8	SAMD00242884
Aw039	A	275	9.04	milk & solid	no	no	133.6	12.5	0.0	5.9	0.0	0.0	0.0	0.0	0.0	4.9	SAMD00242885
Aw040	A	282	9.27	milk & solid	no	no	126.6	0.0	0.0	15.0	0.0	0.0	0.0	0.0	0.0	5.2	SAMD00242886
Aw041	A	289	9.50	milk & solid	no	no	120.2	5.7	0.0	9.2	0.0	0.0	0.0	0.0	0.0	5.3	SAMD00242887
Aw042	A	296	9.73	milk & solid	no	no	110.9	3.5	0.0	13.9	0.0	0.0	0.0	0.0	0.0	5.4	SAMD00242888
Aw043	A	303	9.96	milk & solid	no	no	91.8	12.3	0.0	15.8	0.0	10.5	0.0	0.0	0.0	5.4	SAMD00242889
Aw044	A	310	10.19	milk & solid	no	no	128.1	2.2	0.0	34.4	0.0	0.0	0.0	0.0	0.0	5.2	SAMD00242890
Aw045	A	316	10.39	milk & solid	no	no	96.7	0.0	0.0	37.0	0.0	0.0	0.0	0.0	0.0	5.6	SAMD00242891
Aw046	A	324	10.65	milk & solid	no	no	55.1	0.0	0.0	16.7	0.0	0.0	0.0	0.0	0.0	5.7	SAMD00242892
Aw047	A	331	10.88	milk & solid	no	no	41.6	0.0	0.0	14.8	0.0	0.0	0.0	0.0	0.0	5.3	SAMD00242893
Aw048	A	338	11.11	solid	no	no	42.3	0.0	8.7	22.6	0.0	0.0	0.0	0.0	0.0	5.5	SAMD00242894
Aw049	A	345	11.34	solid	no	no	70.3	0.0	4.0	26.9	3.8	0.0	0.0	0.0	0.0	5.0	SAMD00242895
Aw050	A	352	11.57	solid	no	no	75.4	3.7	25.0	14.9	3.5	5.2	0.0	0.0	0.0	6.7	SAMD00242896
Aw051	A	359	11.80	solid	no	no	91.3	0.0	17.6	24.5	6.0	0.0	0.0	0.0	0.0	6.6	SAMD00242897
Aw052	A	366	12.03	solid	no	no	51.5	0.0	30.0	9.2	2.7	0.0	0.0	0.0	0.0	7.7	SAMD00242898
Aw058	A	402	13.22	solid	no	no	23.5	0.0	31.7	13.1	0.0	0.0	0.0	0.0	0.0	7.4	SAMD00242899
Aw060	A	417	13.71	solid	no	no	53.5	4.4	23.0	6.3	0.0	0.0	0.0	0.0	0.0	6.5	SAMD00242900
Aw062	A	428	14.07	solid	no	no	34.0	0.0	13.9	11.8	0.0	0.0	0.0	0.0	0.0	6.8	SAMD00242901
Aw066	A	457	15.02	solid	no	no	62.0	0.0	0.0	28.8	0.0	0.0	0.0	0.0	0.0	7.8	SAMD00242902
Aw070	A	491	16.14	solid	no	no	64.3	8.2	0.0	20.9	3.9	0.0	0.0	0.0	0.0	7.0	SAMD00242903
Aw074	A	522	17.16	solid	no	no	41.3	0.0	0.0	15.2	0.0	0.0	0.0	0.0	0.0	7.7	SAMD00242904

Only the information of 70 samples were shown here. The value for all 1,070 samples (1,048 samples from 12 infants and 22 samples from 22 adults) are provided in Excel file.
